# High-efficiency dysprosium-ion extraction enabled by a biomimetic nanofluidic channel

**DOI:** 10.1038/s41467-024-50237-9

**Published:** 2024-07-12

**Authors:** Weiwen Xin, Yanglansen Cui, Yongchao Qian, Tianchi Liu, Xiang-Yu Kong, Haoyang Ling, Weipeng Chen, Zhehua Zhang, Yuhao Hu, Lei Jiang, Liping Wen

**Affiliations:** 1 CAS Key Laboratory of Bio-inspired Materials and Interfacial Science, Technical Institute of Physics and Chemistry, Chinese Academy of Sciences, Beijing, 100190 PR China; 2 School of Future Technology, University of Chinese Academy of Sciences, Beijing, 100049 PR China; 3 School of Chemistry and Materials Science, University of Science and Technology of China, Hefei, Anhui 230026 PR China; 4 Suzhou Institute for Advanced Research, University of Science and Technology of China, Suzhou, Jiangsu 215123 PR China; 5 Qingdao Institute of Bioenergy and Bioprocess Technology, Chinese Academy of Sciences, Qingdao, 266101 PR China

**Keywords:** Nanofluidics, Organic-inorganic nanostructures, Bioinspired materials

## Abstract

Biological ion channels exhibit high selectivity and permeability of ions because of their asymmetrical pore structures and surface chemistries. Here, we demonstrate a biomimetic nanofluidic channel (BNC) with an asymmetrical structure and glycyl-L-proline (GLP) -functionalization for ultrafast, selective, and unidirectional Dy^3+^ extraction over other lanthanide (Ln^3+^) ions with very similar electronic configurations. The selective extraction mainly depends on the amplified chemical affinity differences between the Ln^3+^ ions and GLPs in nanoconfinement. In particular, the conductivities of Ln^3+^ ions across the BNC even reach up to two orders of magnitude higher than in a bulk solution, and a high Dy^3+^/Nd^3+^ selectivity of approximately 60 could be achieved. The designed BNC can effectively extract Dy^3+^ ions with ultralow concentrations and thereby purify Nd^3+^ ions to an ultimate content of 99.8 wt.%, which contribute to the recycling of rare earth resources and environmental protection. Theoretical simulations reveal that the BNC preferentially binds to Dy^3+^ ion due to its highest affinity among Ln^3+^ ions in nanoconfinement, which attributes to the coupling of ion radius and coordination matching. These findings suggest that BNC-based ion selectivity system provides alternative routes to achieving highly efficient lanthanide separation.

## Abstract (web summary)

Separation of rare-earth ions is challenging due to their chemical and physical similarities. Here, the authors fabricate a biomimetic nanofluidic channel featuring an asymmetrical structure functionalized with glycyl-L-proline for selective and unidirectional extraction of dysprosium ions.

## Introduction

Rare-earth elements (REEs) are a family of 17 metallic elements, including the lanthanide (Ln) series (La−Lu) and group IIIB (Sc and Y). They are strategic resources with potential applications in catalytic converters, lasers, permanent magnets, and batteries^1–4^. Preconcentration and beneficiation of REEs play critical roles in the production and recycling of REE sources^5^. Traditionally, REE separation is achieved by employing organic solvent-intensive hydrometallurgy techniques with acid-leaching procedures. However, these processes are complex, produce a substantial footprint, and generate environmental pollution from acid and alkali waster liquids. Additionally, access to individual REEs is limited because of the chemical and physical similarities of Ln^3+^ salts during separation. The valence 4*f*-orbital plays little or no role in bonding, leading to minor thermodynamic differences between the binding affinities to certain ligands^6–8^. Meanwhile, Ln contraction decreases the ionic radii across the Ln series by less than 0.2 Å, and the average difference between their values obtained for neighboring elements is only 0.01 Å (Supplementary Table 1). These subtle thermodynamic and dynamic changes make the effective separation of Ln^3+^ ions an extremely challenging task.

**Fig. 1:**
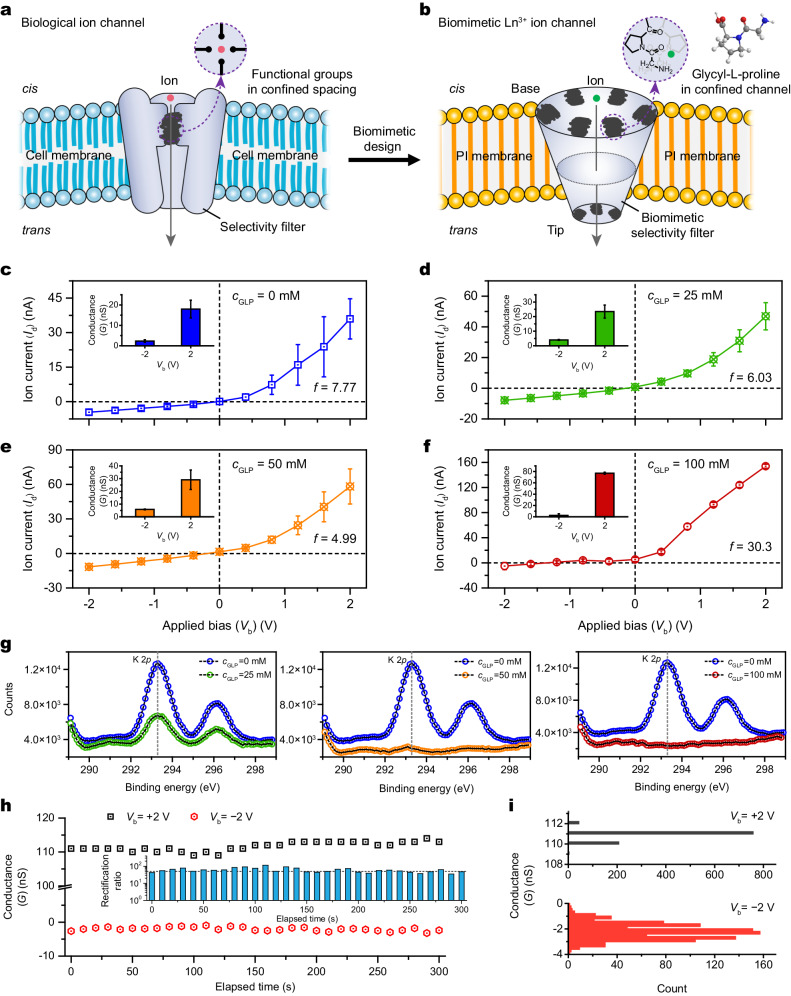
Asymmetric ion transport of the BNC. **a** Biological nanochannel with binding sites in a confined space for selective ion transport. **b** Strategy of constructing the BNC decorated by GLPs. **c**–**f** *I*–*V* curves of the asymmetric ion channel functionalized by using GLPs with concentrations of 0 mM (**c**), 25 mM (**d**), 50 mM (**e**), and 100 mM (**f**), respectively. Ion rectification (*f*) calculated as *f* = |*I*_+2 V_ | / | *I*_−2 V_| demonstrates the enhanced unidirectional ion conduction and ion flux due to the adequate GLP grafting (100 mM). Insets in (**c**–**f**) exhibit the ion conductance of the various channels measured at ±2 V. **g** K 2*p* XPS profiles of the unmodified PI and PI functionalized by using GLPs with different concentrations, such as 0 mM (blue circles), 25 mM (green circles, left plane), 50 mM (orange circles, middle plane), and 100 mM (red circles, right plane), respectively. At a GLP concentration of 100 mM, the typical peak at 293.3 eV assigned to K 2*p* disappears, indicating a high-density grafting of GLPs. **h** Conductance traces of the BNC–GLP_100_ that last for ~300 s at ±2 V. The conductance measured at +2 V is maintained at approximately 115 nS, whereas the conductance measured at −2 V is maintained at approximately 0 nS. Accordingly, the rectification ratio exhibits reproducible cycling performance (inset). **i** Conductance histograms (*n* = 10^3^) at +2 V (top) and −2 V (bottom) showing unidirectional ion conduction, which mimics the transport behavior of uniporters in living organisms. Error bars give the standard deviation from three independent tests.

To date, various approaches to Ln^3+^ ion separation have been developed, including precipitation and crystallization, ion exchange, solvent extraction, and biological adsorption^3,9–20^. Responses to ion radius and chemical affinity can be further amplified in confined nanofluidic channels owing to additional interactions (for example, van der Waals, electrostatic, and orbital interactions)^21^, which enhance ion transport properties, including ion throughput and selectivity. These characteristics make nanofluidic channels promising platforms for Ln^3+^ ion extraction with high separation efficiency. For instance, biological potassium channels include protein pores in cell membranes with ultrahigh K^+^ ion conductivity and selectivity compared with those of other ions with similar sizes and the same valence. The K^+^ permeance is at least 10^4^ times greater than that of Na^+^ ions, despite a difference in ionic radii of only 0.38 Å (ref. ^22^). Such excellent ion selectivity is mainly derived from subnanometer-sized and flexible ion selectivity filters, while the ultrahigh and unidirectional ion permeability originates from the asymmetrical pore structure and surface chemistry (Fig. 1a). This inspired us to construct artificial nanochannels with selective transport of Ln^3+^ ions by mimicking biological ion channels. Recently, some bacteria have demonstrated the selectivity and fractionation for heavy REEs owing to the specific functional groups in cells^14,23^. In particular, a highly selective Ln^3+^-binding protein, called lanmodulin (LanM), was found in bacteria that use lanthanides, and it offers a promising avenue for Ln^3+^ ion separation^1,16,24–26^. The extraordinary proline residues of LanM contribute to the selective chelation of Ln^3+^ ions^16^; therefore, they may serve as binding sites for biomimetic Ln^3+^ ion channels.

Herein, we report a biomimetic nanofluidic channel (BNC) for selective Ln^3+^ ion extraction that exhibits a remarkable heavy/light REE (HREE/LREE, see Supplementary Table 2) separation efficiency with high-performance Dy^3+^/Nd^3+^ selectivity of approximately 60. The asymmetrically structured BNC functionalized with glycyl-L-proline (BNC–GLP) can rapidly and unidirectionally transport Ln^3+^ ions across the BNC, and the resulted conductivities in the BNC reach up to two orders of magnitude higher than in a bulk solution. Even in a mixture containing six Ln^3+^ ions in solution, the BNC–GLP retains its high performance for selective Dy^3+^ transport over other Ln^3+^ ions, overcoming the disadvantage of severe competitive permeation. In a Nd^3+^-solution containing an ultralow concentration of Dy^3+^ ions (3 wt.%), the biomimetic Ln^3+^ ion channel preferentially transfers Dy^3+^ and purifies Nd^3+^ (99.8 wt.%) ions, achieving the further beneficiation of Dy^3+^ ions in the mixed solution.

## Results

### Fabrication and characteristics of BNC–GLP

Inspired by biological ion channels (Fig. 1a), we constructed inventively a biomimetic Ln^3+^ ion channel with enhanced target separation performance in this work (Fig. 1b). We fabricated a single conical nanochannel embedded in a commercial polyimide (PI) membrane using a well-developed asymmetric track-etched methodology^27,28^ (Supplementary Fig. 1). The obtained diameters of the base and tip were approximately 750 and 10 nm, respectively (Supplementary Fig. 2). Notably, the polyester PI material is suitable for generating a charged surface containing carboxylate groups with a density of approximately 1.5 groups nm^−2^, which is beneficial for the subsequent functionalization and modification processes. We combined GLP and the aforementioned single conical PI nanochannel to assemble a functionalized ion channel with ion conduction in response to Ln^3+^ ions based on the association between Ln^3+^ ions and GLP in nanoconfined systems.

We used the experimental set-up (Supplementary Fig. 1d) to examine the surface charge-governed ion transport, which is influenced by the concentration of GLP on the inner surface. The degree of functionalization can be demonstrated by plotting current−voltage (*I*–*V*) curves at the symmetrical KCl electrolytes. The unmodified nanochannel exhibits ion rectification (*f*) of 7.77, as shown in Fig. 1c. This value is calculated based on the ratio of ion current (*I*_d_) at +2 V and −2 V, respectively. By contrast, the nanochannels modified by 25 and 50 mM GLP (*c*_GLP_) demonstrate lower *f* values due to the increased *I*_d_ at negative biases (Fig. 1d, e). A noteworthy shift of *f* from 6.03 to 30.3 arises from the substantial decrease in *I*_d_ at negative biases and the enormous increase in *I*_d_ from approximately 60 to 160 nA at positive biases through the nanochannel modified by 100 mM GLP, greatly contributing to unidirectional, ultrafast, and high-flux ion transport (Fig. 1f). Additionally, at a GLP concentration of 150 mM (Supplementary Fig. 3a), *f* decreases to 4.38 because the partial electrostatic assembly of GLPs in nanoconfinement blocks the functional channel. X-ray photoelectron spectroscopy (XPS) was employed to investigate the modification degree of deprotonated carboxylic groups on the surface of the nanochannel. As shown in Fig. 1g, the relative intensity of K 2*p* decreases as the GLP concentration increases from 25 to 100 mM, indicating the gradually grafting of GLPs. Excessively increasing the GLP concentration produces a minimal effect on the modification degree (Supplementary Fig. 3b). Accordingly, the estimated GLP coverage is nearly 100%, which is equivalent to the density of almost 1.5 GLPs nm^−2^, rendering the high selectivity for certain ions. Accordingly, the system showed excellent stability with steady ion conductance at +2 V and −2 V (Fig. 1h). The corresponding rectification of BNC–GLP_100_ (here, 100 refers to the GLP concentration equal to 100 mM) reached up to ~40 (inset of Fig. 1h). Additionally, the conductance histograms (Fig. 1i), including 1000 statistical points, further confirm the robust system and repeatable transport behaviors. In this case, the GLP could even maintain structural integrity in acid solutions for one week (Supplementary Fig. 4).

### The amplified Ln^3+^ ion transport differences in BNC–GLP_100_

**Fig. 2:**
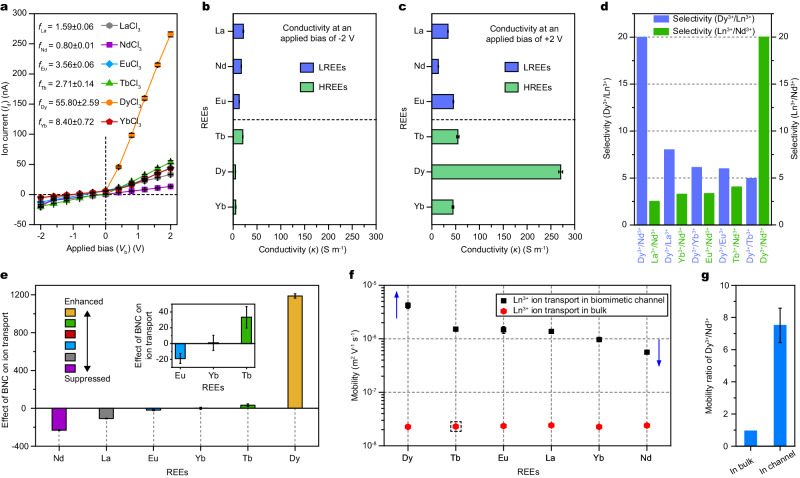
The amplified transport differences of Ln^3+^ ions and ultrafast Ln^3+^ ion transport in the BNC–GLP_100_. **a** *I*–*V* curves of REEs across the BNC–GLP_100_ measured in 0.1 M electrolyte solutions (pH 4.0). **b**, **c** Ion conductivities (*κ*) of Ln^3+^ ions across the BNC–GLP_100_ obtained at ±2 V. The ion conductivities measured at +2 V are substantially higher than those determined at −2 V (**b**), and the ion conductivities of LREEs are significantly lower than HREEs (**c**). **d** Selectivities (*S*) of Dy^3+^/Ln^3+^ and Ln^3+^/Nd^3+^ passing across the BNC–GLP_100_ at +2 V, calculated as *S* = *G*_Dy_/*G*_REEs_ and *S* = *G*_REEs_/*G*_Nd_, respectively. **e** Effect of BNC–GLP_100_ on Ln^3+^ ion transport, defined as the normalized net current. The inset depicts the transition from the enhanced to suppressed states. **f** Ln^3+^ ion mobilities in the BNC–GLP_100_ (black squares) and the bulk solution (red hexagons). The mobilities in the BNC–GLP_100_ are higher than the corresponding bulk values by 100 times due to the ultrafast ion conduction in nanoconfinement. The mobility of Tb^3+^ ions in the bulk solution is an estimated range. **g** Experimental Ln^3+^ ion mobility ratios obtained in the bulk solution and BNC, respectively. Error bars give the standard deviation from three independent tests.

*I*–*V* curves were recorded for a series of Ln^3+^ chlorides to investigate their transport properties in the nanoconfined channel. As shown in Fig. 2a, all the obtained curve profiles are asymmetrically parabolic. Specifically, the *I*_*d*_ values at +2 V for DyCl_3_ and NdCl_3_ are 2.66 × 10^2^ and 13.3 nA, respectively, corresponding to the largest and smallest values among the six Ln^3+^ ions. Correspondingly, the ion flux of DyCl_3_ (~5.52 × 10^11^ ions s^−1^) is approximately one order of magnitude higher than that of NdCl_3_ (~2.76 × 10^10^ ions s^−1^) across BNC–GLP_100_, respectively, along with the greatly different rectification ratios of 55.7 and 1.25. In addition, the calculated REE rectification ratios are in the order of *f*_Dy_ (55.80 ± 2.59) > *f*_Yb_ (8.40 ± 0.72) > *f*_Eu_ (3.56 ± 0.06)~*f*_Tb_ (2.71 ± 0.14) > *f*_La_ (1.59 ± 0.06)~*f*_Nd_ (0.80 ± 0.01). Furthermore, the corresponding BNC–GLP_100_ conductivity (*κ*) values obtained at +2 V are ranked in the order of *κ*_Dy_ (270.6 ± 4.2 S m^−1^) > *κ*_Tb_ (54.6 ± 2.5 S m^−1^) > *κ*_Yb_ (44.2 ± 1.8 S m^−1^)~*κ*_Eu_ (45.2 ± 0.7 S m^−1^) > *κ*_La_ (33.8 ± 0.3 S m^−1^) > *κ*_Nd_ (13.5 ± 0.3 S m^−1^) (Fig. 2b). These magnitudes are significantly different from the values measured at a bias of −2 V (Fig. 2c). Note that the observed differences in the ion conductivity are mainly caused by the Ln^3+^ ions, because the salt solutions shared the same anion (Cl^−^). Accordingly, the Dy^3+^/Nd^3+^, Dy^3+^/La^3+^, Dy^3+^/Yb^3+^, Dy^3+^/Eu^3+^, and Dy^3+^/Tb^3+^ selectivities of BNC–GLP_100_ at +2 V calculated from the corresponding *κ*_Dy_/*κ*_REEs_ ratios are 19.99, 8.00, 6.12, 5.99, and 4.95, respectively, whereas other Ln^3+^/Nd^3+^ selectivities are below 4.00 (Fig. 2d).

To further characterize the amplified Ln^3+^ ions transport differences across BNC–GLP_100_, the normalized net currents, denoted as (*I*_d_‒*I*_b_)/*I*_min_ (“Methods” and Supplementary Fig. 5), signifying the effect of BNC–GLP_100_ on ion transport were calculated (Fig. 2e). The *I*_d_ of Yb^3+^ through BNC–GLP_100_ with the smallest value is set to *I*_min_, and the ion current of Ln^3+^ ions through the unmodified nanochannel is set to *I*_b_. For Tb^3+^ and Dy^3+^ ions, BNC–GLP_100_ enhances the transmembrane ion transport with positive (*I*_d_‒*I*_b_)/*I*_min_, and for Dy^3+^ ion in particular, its transport capacity is increased by 1200 times. In contrast, BNC–GLP_100_ suppresses the transport of Nd^3+^, La^3+^, and Eu^3+^ ions, particularly Nd^3+^ ion, by more than 200 times. To gain more insight into the influence of nanoconfinement on ion transport, we conducted drift-diffusion experiments and measured the conductivities of various chloride solution. The final mobilities of Ln^3+^ ions in BNC–GLP_100_ and the bulk solutions are plotted in Fig. 2f. The mobilities in the bulk solutions with slight variations calculated for different Ln^3+^ ions are 2.2–2.4 × 10^−8^ m^2^ V^−1^ s^−1^. Meanwhile, the mobilities in BNC–GLP_100_ increase by a factor of 10–421.6 × 10^−8^ and 56.2 × 10^−8^ m^2^ V^−1^ s^−1^ for Dy^3+^ and Nd^3+^ ions, respectively. The mobilities in BNC–GLP_100_ are nearly two orders of magnitude higher than those in the bulk solutions, demonstrating ultrafast transport properties (Supplementary Table 3). Importantly, the mobility ratio of Dy^3+^/Nd^3+^ measured in BNC–GLP_100_ is approximately eight times higher than the bulk value (Fig. 2g). These results reveal that the biomimetic nanofluidic Ln^3+^ ion channel significantly amplifies the differences between various Ln^3+^ ions due to nanoconfined transport and interaction.

### Selective interactions between GLP and Ln^3+^ ions

**Fig. 3:**
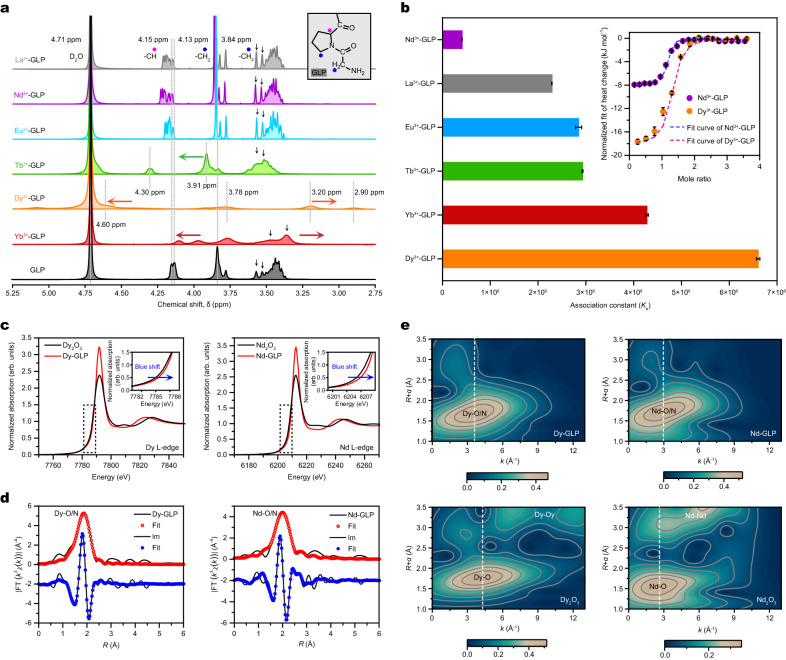
Evaluation of the selective interactions between Ln^3+^ ions and GLPs. **a** Partial NMR analyses of GLP showing the proton resonance shifts obtained for different Ln^3+^ ions in D_2_O (4.71 ppm) with TMS, used as an internal standard set to 0 ppm. The additions of Ln^3+^ ions lead to different shifts with respect to the reference peak (GLP, black line). The concentrations of GLPs and Ln^3+^ ions in D_2_O are set to 2 and 0.2 mM, respectively. The inset shows the structure of GLP with H signals from −CH_2_ species located at 3.84 and 4.13 ppm (blue spots) and from −CH species located at 4.15 ppm (pink spots). **b** Association constants (*K*_a_) of Ln^3+^ ions with GLPs derived by fitting the corresponding ITC curves. Insert: variations in the observed heat changes plotted against the molar ratios of Nd^3+^ and Dy^3+^ ions to GLPs, respectively, determined by titrating the Ln^3+^ solutions into the GLP solutions. **c** Normalized L_3_-edge XANES spectra of Ln_2_O_3_ and Ln−GLP. Insets: magnified partial XANES spectra showing slight blue shifts. **d** Ln L_3_-edge XANES spectra of Ln_2_O_3_ and Ln−GLP. Fourier transform−EXAFS spectra fittings for Ln-GLP at Dy L_3_-edge (left) and Nd L_3_-edge (right). *R* (Å) is the peak location scale used for the Fourier-transformed magnitude in real space. **e** WT–EXAFS Ln L_3_-edge spectra recorded for Dy−GLP, Nd−GLP, and their references. The vertical dashed lines are drawn to guide the eye. *α* denotes the phase shift. Metallic bonds, such as Dy−Dy and Nd−Nd, are observed in the reference samples and not in the Ln−GLP samples. Error bars give the standard deviation from three independent tests.

The interactions between GLP and different Ln^3+^ ions were examined by measuring ^1^H nuclear magnetic resonance (NMR) signal variations of GLP in the presence of various Ln^3+^ ions in deuterium oxide solvent (Fig. 3a). A comparison of the obtained spectra revealed that upon exposure to Ln^3+^ ions, the related GLP proton resonances (e.g., at 4.15, 4.13 and 3.84 ppm) exhibited distinct downfield shifts with respect to that of pure GLP. These shifts could be attributed to the shielding effect of the coordinating donor atom electron density of GLP around Ln^3+^ ions^29^, implying the chelation between GLP and Ln^3+^ ions. Note that these ^1^H signal changes can be explained by the pseudocontact shifts (PCSs) that stem from through-space interactions with the unpaired electrons in orbitals of the paramagnetic center^30^ (Supplementary Table 4). In this regard, diamagnetic La^3+^ ions exhibited negligible shifts (Fig. 3a), acting as a reference for estimating the difference of PCSs. Accordingly, the nonisotropic environments of unpaired electrons (4*f*-orbitals) enable a short electronic relaxation time of paramagnetic Ln^3+^ ions, thereby promoting low-lying excited states that cause strong magnetic anisotropy and fast electron relaxation. Thus, these results lead to prominent PCSs and less ^1^H relaxation enhancement. Figure 3a shows that some regenerative, split, and nearly disappeared ^1^H NMR signals from protons of GLP binding Dy^3+^ ions are detected, whereas their PCSs are measurable at a scale of several nanometers^30,31^. These shifts indicate that there are structural changes near the center of the lanthanide series which may result from the decreased Ln^3+^ ion size and the strict matching requirements of the GLP. More importantly, the different chemical shifts suggest that the heavy Ln^3+^ ions can better match with GLP compared to light Ln^3+^ ions, thereby resulting in the formation of more kinetically rigid structures^32^. In contrast, less paramagnetic Nd^3+^ ions exhibit smaller PCSs and weak relaxation, which are also observed for alkali and alkaline-earth metal ions (Supplementary Fig. 6). These differences in electronic shells of Ln^3+^ ions around binding sites of GLP may cause different affinities in thermodynamics and dynamics. Furthermore, isothermal titration calorimetry (ITC) was performed to quantify the intermolecular interaction strength between GLP and Ln^3+^ ions. By integrating the raw titration profiles (Supplementary Fig. 7), the normalized heat changes with increasing mixing molar ratios of Ln^3+^ ions to GLP were presented (Supplementary Fig. 8). As shown in Fig. 3b, the heat change curves demonstrated similar changing trends for various Ln^3+^ ions interacting with GLP. However, the changing amplitude and slope are different, which reflect the different interaction strengths. By fitting the heat change curves, the association constants (*K*_a_) of GLP with Ln^3+^ ions are obtained, which decrease in the order of ${K}_{{{\mbox{a}}}}^{{{\mbox{Dy}}}}$ (6.61 × 10^6^ M^−1^) > ${K}_{{{\mbox{a}}}}^{{{\mbox{Yb}}}}$ (4.28 × 10^6^ M^−1^) > ${K}_{{{\mbox{a}}}}^{{{\mbox{Tb}}}}$ (2.93 × 10^6^ M^−1^) > ${K}_{{{\mbox{a}}}}^{{{\mbox{Eu}}}}$ (2.85 × 10^6^ M^−1^) > ${K}_{{{\mbox{a}}}}^{{{\mbox{La}}}}$ (2.29 × 10^6^ M^−1^) > ${K}_{{{\mbox{a}}}}^{{{\mbox{Nd}}}}$ (4.11 × 10^5^ M^−1^). Due to the high-precise experimental instrument and strict operation condition control, the uncertainties of the final *K*_a_ values are well limited in less than 3% (Fig. 3b). Consequently, the *K*_a_ of Dy^3+^ is approximately 16 times higher than that of Nd^3+^, suggesting the strongest interactions between Dy^3+^ and GLP species (see insert in Fig. 3b).

X-ray diffraction (XRD) and XPS patterns confirmed the retention of the GLP structure and distinct signals of Ln^3+^ ions (Supplementary Figs. 9 and 10). To further verify the selective interactions between GLP and Ln^3+^ ions, we studied the changes in the local electronic and atomic structures of the chelates composed of Ln^3+^ ion and GLP by applying X-ray absorption spectroscopy (XAS), including X-ray absorption near edge structure (XANES) and extended X-ray absorption fine structure (EXAFS) techniques^15,33^. Figure 3c and Supplementary Figs. 11−14 display the Ln L_3_-edge XAS spectra of Ln_2_O_3_ and Ln−GLP. In particular, the XANES spectra show the presence of Dy L_3_-edge and Nd L_3_-edge at 7791.8 and 6212.6 eV, respectively, indicating that the valence states (+3) of Dy and Nd are unaltered. Further analyses revealed slight blue shifts in their XANES spectra compared with those of Ln_2_O_3_ compounds, which resulted from the corresponding changes in the coordination environments. The fitting of the obtained EXAFS spectra produced the Dy–O/N and Nd–O/N bond lengths of approximately 2.36 ± 0.01 and 2.49 ± 0.02 Å, respectively (Fig. 3d and Supplementary Table 5), and the corresponding coordination numbers were 8.7 ± 0.7 (Dy−O/N) and 8.6 ± 1.8 (Nd−O/N). The EXAFS spectra and their fitting parameters obtained for other Ln−GLP composites are also displayed in Supplementary Figs. 11−14 and Supplementary Table 6.

To reveal the underlying coordination environments, wavelet transform (WT) analyses of the EXAFS spectra were performed to determine both *R*-space and *k*-space information. Their results confirmed the absence of a secondary correlation with the scattering of binuclear or cluster [Ln···Ln] species, such as [Dy···Dy] and [Nd···Nd] in *R*-space. The estimated [Dy···Dy] and [Nd···Nd] distances in Dy_2_O_3_ and Nd_2_O_3_ were equal to 3.4 and 3.7 Å, respectively (Fig. 3e and Supplementary Figs. 15 and 16). Furthermore, the WT maximum determined for Dy−O/N (3.6 Å^−1^) exhibited a lower *k*-value than that of Dy−O (4.35 Å^−1^), suggesting the presence of coordinated N and O atoms. In contrast, the change in the *k*-values of Nd–O (2.56 Å^−1^) and Nd–O/N (2.96 Å^−1^) is subtle, indicating that less N and O atoms of GLP participate in chelating Nd^3+^ ions. Note that the appreciable downfield shifts of *k*-values in other Ln−O/N configurations were observed in GLP compounds compared with their Ln_2_O_3_ samples (Supplementary Fig. 17). Overall, the observed variation in the coordination number in the GLP-modified nanochannel, as compared to the bulk solution, implies that GLP offers supplementary binding sites for Dy^3+^ ions, thereby facilitating the formation of more stable chelates between Dy^3+^ ions and GLP.

### Highly efficient Ln^3+^ ion extraction enabled by BNC–GLP_100_

**Fig. 4:**
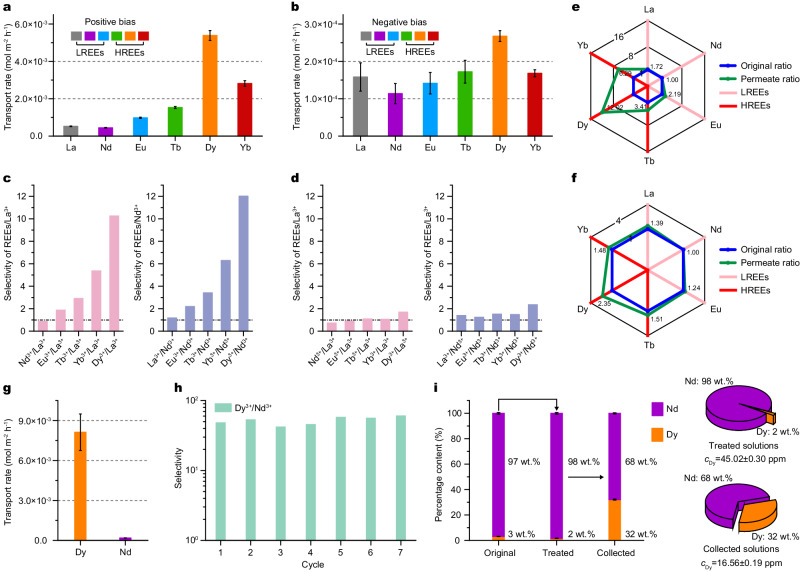
Separation performance of Ln^3+^ ions in the BNC–GLP_100_. **a**, **b** REE transport rates measured by using ICP–MS under the applied constant positive (**a**) and negative (**b**) biases. BNC–GLP_100_ can rapidly transport REEs at a +2 V bias and transport rates of up to one order of magnitude higher than those obtained at a −2 V bias. **c**, **d** Ln/La and Ln/Nd selectivities obtained at +2 V (**c**) and −2 V (**d**). **e**, **f** Radial plots of the Ln^3+^ ion concentration ratios measured for the original and permeate solutions at +2 V (**e**) and −2 V (**f**) biases, respectively. The Ln^3+^ ion concentrations in the original solutions are the same. **g** Dy^3+^/Nd^3+^ selectivity of the BNC–GLP_100_ in the binary solution. **h** Cycling performance of Dy^3+^/Nd^3+^ selectivity in the binary ion solution. **i** Separation of Dy^3+^ and Nd^3+^ ions in the high-concentration neodymium system with a Nd^3+^/Dy^3+^ ratio of 32.3/1 (wt.%). Selective ion diffusion is conducted by applying a constant potential of +2 V across the BNC–GLP_100_ to achieve the target Nd^3+^ concentration in the treated solutions and Dy^3+^ extraction in the permeate solutions, respectively. Error bars give the standard deviation from three independent tests.

The dependence of the ion transport rate on external bias was also investigated by using a picoammeter and inductively coupled plasma mass spectrometry (ICP–MS) at an applied constant potential of +2 V or −2 V (Supplementary Fig. 18). The transport rates of Ln^3+^ ions measured at a constant +2 V bias are presented in Fig. 4a and Supplementary Fig. 19a. Their values are approximately one order of magnitude higher than those recorded at a constant bias of −2 V (Fig. 4b and Supplementary Fig. 19b). More importantly, the transport rates of HREEs are greater than those of LREEs at a +2 V bias, whereas their differences are negligible at a −2 V bias (Fig. 4c, d and Supplementary Fig. 19c). This can be attributed to the enhanced migration of REEs and effective unidirectional transport behaviors at a positive bias^27,34^. Note that BNC–GLP_100_ enables highly efficient Dy^3+^ transport at a rate of 5.39 × 10^−3^ mol m^−2^ h^−1^. In a mixture containing six Ln^3+^ ions with the same concentrations, BNC–GLP_100_ successfully achieve the preconcentration and separation of Dy^3+^ ions, and the permeation ratio obtained at a constant +2 V bias is equal to 12.02 (Dy): 6.29 (Yb): 3.41 (Tb): 2.19 (Eu): 1.72 (La): 1.00 (Nd) (Fig. 4e). Correspondingly, the permeation ratio obtained at a constant −2 V bias is 2.35 (Dy): 1.51 (Tb): 1.48 (Yb): 1.39 (La): 1.24 (Eu): 1.00 (Nd), respectively (Fig. 4f).

Consequently, we performed the Dy^3+^/Nd^3+^ separation by using BNC–GLP_100_ for showing the potential in recycling Dy^3+^ resource from a high-concentration Nd^3+^ solution^10,35,36^. The permeation rates in the binary mixture solution of Dy^3+^ and Nd^3+^ ions were determined to be 8.13 × 10^−3^ and 1.76 × 10^−4^ mol m^−2^ h^−1^, respectively, which corresponded to a separation ratio greater than 46 (Fig. 4g and Supplementary Fig. 20). With relatively large difference between Dy^3+^ and Nd^3+^ ions and less competitive species for the transport, the BNC–GLP_100_ presented a much higher Dy^3+^/Nd^3+^ selectivity in the binary mixture solution. Additionally, after seven cycles of Dy^3+^/Nd^3+^ separation measurements, the separation ratio remained at above 60 (Fig. 4h), exhibiting the remarkable cycling performance of BNC–GLP_100_. To explore the ability of BNC–GLP_100_ to extract Dy^3+^ ions from highly concentrated Nd^3+^ solutions, the original solutions with a Nd/Dy weight ratio of 32.3/1 were used (Fig. 4i). After a constant potential of +2 V was applied for 48 h, the Nd^3+^ content increased to 98 wt.%, and the Dy^3+^ content decreased to only 2 wt.% (45 ppm) in the treated solutions. The collected solutions contained up to 32 wt.% Dy^3+^, corresponding to 17 ppm extracted from the highly concentrated Nd^3+^ solutions (Fig. 4i and Supplementary Fig. 21). We repeated the separation process for five cycles, obtaining almost pure Nd^3+^ (99.8 wt.%) solutions (Supplementary Fig. 22). The Dy^3+^ concentration is decreased from 3.1 wt.% to 0.2 wt.%, with Nd^3+^ concentration increasing from 96.9 wt.% to 99.8 wt.% in the feeding solution. The proposed method is sustainable and free of organic reagents and, thus, advantageous for targeted separation, making the recycling of these critical materials economically feasible^37^.

**Fig. 5:**
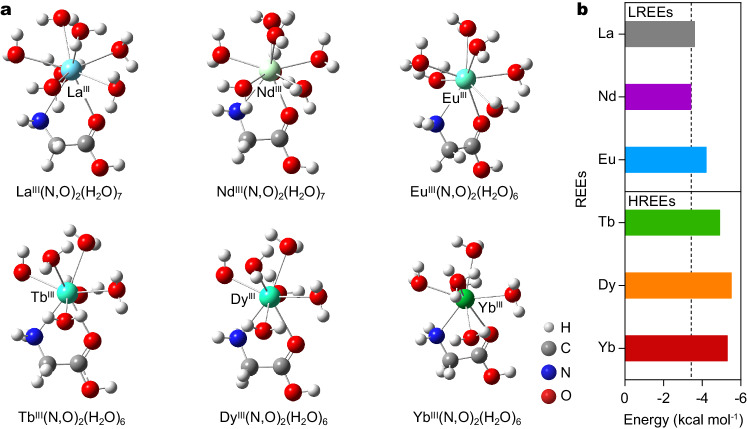
Theoretical computations for the interactions between Ln^3+^ ions and GLP. **a** Optimized geometries of Ln^3+^ ions and GLPs in water. The gray, white, blue, and red spheres represent C, H, N, and O atoms in GLP, and O atoms in water, respectively. The green-to-blue gradient spheres denote Ln^3+^ ions (La^3+^, Nd^3+^, Eu^3+^, Tb^3+^, Dy^3+^, and Yb^3+^). By coordinating with GLP molecules, Ln^3+^ ions lose two water molecules to form steady complexes. In these complexes, the numbers of hydrated water molecules for La^3+^ and Nd^3+^ ions are 7; the number of hydrated water molecules for Eu^3+^, Tb^3+^, Dy^3+^, and Yb^3+^ ions are 6. **b** Energies of formation determined for the complexes of Ln^3+^ ion and GLP with the coordinated water molecules. The energy values obtained for LREEs are considerably lower than those determined for HREEs. The binding energies of Ln^3+^ ions can be ranked in the order of Dy^3+^ (−5.5 kcal mol^−1^) > Yb^3+^ (−5.3 kcal mol^−1^) > Tb^3+^ (−4.9 kcal mol^−1^) > Eu^3+^ (−4.2 kcal mol^−1^) > La^3+^ (−3.6 kcal mol^−1^) > Nd^3+^ (−3.4 kcal mol^−1^).

To elucidate the selectivity of Ln^3+^ ions in BNC–GLP_100_, we calculated the relative binding energies of Ln^3+^ ions to GLP by using a simplified model that contained a GLP molecular and a single ion. By coordinating with a GLP molecule, Ln^3+^ ions lose two water molecules to form steady complexes (Supplementary Tables 7−12), and thus Fig. 5a shows the presence of Ln^3+^ and GLP composites with two different forms: hexahydrate [Eu^III^(N,O)(H_2_O)_6_, Tb^III^(N,O)(H_2_O)_6_], [Dy^III^(N,O)(H_2_O)_6_], and [Yb^III^(N,O)(H_2_O)_6_], and heptahydrate [La^III^(N,O)(H_2_O)_7_, and Nd^III^(N,O)(H_2_O)_7_]. Hence, Ln^3+^ ions can be anchored in the form of Ln^3+^···O and Ln^3+^···N species with different binding energies. As shown in Fig. 5b, the binding energies of HREEs are considerably higher than those of LREEs, and the binding energy of Dy^3+^ is −5.5 kcal mol^−1^, which exceeds the values obtained for Yb^3+^ and Tb^3+^ ions. Meanwhile, the binding energy of Nd^3+^ is −3.4 kcal mol^−1^, which is lower than the values determined for La^3+^ and Eu^3+^ ions.

Furthermore, in order to gain insights into the selectivity mechanism in BNC–GLP_100_, we conducted additional transport experiments involving other heavy Ln^3+^ ions (Supplementary Fig. 23) and performed the theoretical calculations to visually demonstrate the interactions (Supplementary Fig. 24). Compared to light Ln^3+^ ions with larger radius sizes, the heavy Ln^3+^ ions exhibited faster transport rates due to their smaller ion radius sizes. Specifically, in case of heavy Ln^3+^ ions, there may be a coordination matching wherein GLP cannot adequately wrap around the smaller Ln^3+^ ions, leading to a drop in stability and association interactions.^32,38^ In this regard, Dy^3+^ ion exhibited a fine binding to GLP, resulting in the formation of a rigid structure, which is confirmed by NMR spectra and computational results (Fig. 3a and Supplementary Fig. 24). Based on these findings, we propose that the size of ion radius and coordination matching play the crucial role in facilitating rapid ion transport, thereby enabling the selective and ultrafast transport of Dy^3+^ ions through BNC–GLP_100_.

## Discussion

A BNC with ultrahigh Dy^3+^ ion selectivity and high performance of Dy^3+^ extraction was successfully fabricated in this study. As a proof of concept, we highlighted that the subtle differences in Ln^3+^ ions with intrinsic chemical similarities were significantly amplified in the confined nanofluidic channel decorated with GLP. Beneficial from the nanoconfined channel in BNC, the coupling of ion radius and coordination matching contributes to the amplified affinity differences between Ln^3+^ ions and BNC–GLP, and the proposed system provides a promising solution to the long-standing challenge of Ln^3+^ ion separation. Specifically, with the BNC–GLP_100_, a separation ratio of Dy^3+^/Nd^3+^ above 60 could be reached. The recycling of consumer materials containing Ln^3+^ ions is a valuable source of various elements, especially Tb^3+^, Dy^3+^, Eu^3+^, Nd^3+^, and Y^3+^, owing to their supply vulnerability and scarcity of clean energy technologies^17,39^. The results of this work further indicated that BNCs are promising for Ln^3+^ ion separation and extraction routes and are advantageous in terms of eco-friendliness, sustainability, and efficiency.

## Methods

### Fabrication of the biomimetic nanofluidic channel

A single conical nanochannel embedded in the PI membrane was prepared by an asymmetric track etching technique. Prior to etching, both sides of the PI membrane were exposed to UV light for 1 h. One side of the membrane was in contact with the etching solution (14 wt.% NaClO), while the other side was in contact with a stop solution (1 M KI). The etching process was conducted at a temperature of 60 °C and applied voltage of +1 V and stopped at a current value corresponding to a certain tip diameter. Subsequently, the etching solution was removed, and the two chambers were filled with the stop solution for 30 min. Finally, the etched PI membrane was immersed in distilled water for the subsequent modifications. The base diameter (*d*_base_) of the single conical PI nanochannels was approximately 750 nm, and the tip diameter (*d*_tip_) can be expressed by the following formula:

1 $${d}_{{tip}}=\frac{4{IL}}{\pi {\kappa }_{c}U{d}_{{base}}}$$

where *κ*_c_ is the specific conductivity in a 1 M KCl solution at 298 K, which is equal to 0.11173 Ω^−1^ cm^−1^. Thus, the estimated *d*_tip_ value was 10 nm.

To modify the single conical nanochannel by using GLPs, the PI membrane was immersed in an aqueous solution of 1-ethyl-3-(3-dimethylaminopropyl)carbodiimide (15 mg mL^−1^) and *N*-hydroxysulfosuccinimide (3 mg mL^−1^) for 1 h. The treated membrane was immersed in an aqueous solution of GLPs with a concentration of 25, 50, 100, or 150 mM at 298 K for 12 h for covalent surface coupling, respectively. Finally, the as-prepared membranes were cleaned with Milli-Q water.

### Characterization techniques

Field-emission scanning electron microscopy (S-4800, Hitachi) coupled with second electron (SE) imaging was used to observe the sample structure and size at an accelerating voltage of 10 kV. XRD measurements were performed on a Micromeritics Tristar II 3020 gas adsorption analyzer combined with a PANalytical B.V. Empyrean powder diffractometer using Cu-K*α* radiation over a range of 2*θ* = 4.0~40.0° at a voltage of 40 kV, current of 40 mA, and step size of 0.02° (2 s per step). ITC (TA NANO) assays were used to examine the interactions between Ln^3+^ ions and GLP. For this purpose, Ln^3+^ ions (0.01~0.02 mmol L^−1^) and GLP (0.001 mmol L^−1^) were respectively dissolved in water (pH 4). GLP was injected into metal-ion-laden sample cells. The control tests were performed by injecting various Ln^3+^ ion solutions (pH 4) into water (pH 4), as shown in Supplementary Fig. 7 (blue lines). For each measurement, the consecutive 20 injections with 200-s intervals between two injections were executed at 25 °C. A series of thermodynamic parameters, including binding constants (*K*_a_), binding ratio (*n*), binding enthalpy (Δ*H*_a_), binding entropy (Δ*S*_a_), and binding free energy (Δ*G*_a_), were obtained using the NanoAnalyze software (Supplementary Table 13). Before fitting, the binding data has been corrected by subtracting the control data. All experiments were carried out in triplicate. The ^1^H NMR spectra were recorded on a Bruker DM300 or AV 400 spectrometer using tetramethylsilane (TMS) as an internal standard. Chemical shifts were quoted in parts per million (ppm) relative to the signals corresponding to residual non-deuterated protons in NMR solvents. ^1^H NMR measurements of GLP (5 mM) were performed in the presence of various metal ions (20 mM) in D_2_O solution to investigate GLP changes (D_2_O: *δ* 1.56 ppm). The EXAFS spectra (Ln L_3_-edge) were obtained at the 4B9A beamline of the Beijing Synchrotron Radiation Facility (BSRF). The BSRF storage rings were operated at 2.5 GeV with a stable current of 400 mA. Using a Si (111) double-crystal monochromator, data collection was conducted in the fluorescence mode using a Lytle detector. All spectra were collected under ambient conditions. Data reduction, data analysis, and EXAFS fitting were performed according to standard procedures using the ATHENA and ARTEMIS software programs included into the Demeter package. Energy calibration was conducted using a standard sample, which also served as a reference. For EXAFS modeling, *k*2-weighted EXAFS spectra were obtained by subtracting the post-edge background from the overall absorption intensity following by normalization with respect to the edge-jump step and Fourier transformation to the real *R*-space using the Hanning window of 1.0–8.0 Å^−1^ (d*k* = 1.0 Å). The obtained EXAFS spectra were fitted to determine the coordination numbers for the Ln−O/N scattering paths.

### *I*–*V* measurements

The ion transport behaviors of both the unmodified single conical PI nanochannel and BNC–GLP membranes were investigated by recording their *I*–*V* curves. Ion currents were measured by using a Keithley 6487 picoammeter (Keithley Instruments, Cleveland, OH) with an apparatus comprising two polymethyl methacrylate (PMMA) chambers, which were separated by the studied membrane. The two chambers were filled with metal ion chloride salt solutions at the same concentration. Homemade Ag/AgCl electrodes were used in each chamber to apply an electric potential across the asymmetrical PI or BNC–GLP membrane. The transmembrane potential utilized in this study was a scanning voltage varied from −2 to +2 V at a step of 0.4 V. The pH values of the electrolyte solutions were adjusted by using 0.1 or 0.01 M HCl solutions. *I*–*V* measurements were performed at 298 K. Each test was repeated at least 11 times to obtain the average current values at different voltages. All the pH values of the used Ln^3+^ solutions were calibrated to 4 by using hydrogen chloride (or deuterium chloride) solution.

### Ion permeation experiments

The BNC–GLP membrane was clamped between two PMMA compartments. One cell facing the base of the BNC–GLP was filled with a mixture of ion solutions containing 10 mL of 0.1 M LnCl_3_ (the mixed six-type Ln^3+^ ion solution comprised LaCl_3_, NdCl_3_, EuCl_3_, TbCl_3_, DyCl_3_, and YbCl_3_ with the same concentrations and the binary solution was composed of equal concentrations of NdCl_3_ and DyCl_3_) used as the feed solution, while the other cell was filled with 10 mL of deionized water serving as the permeate solution. Ion selectivity measurements were conducted by applying a constant bias of +2 V via a Keithley 6487 picoammeter across the BNC − GLP membrane for 48 h using Pt electrodes with both compartments. The set-up for the ion selectivity measurement was entirely sealed to avoid a reaction with CO_2_ from the air. At the end of these experiments, the ion concentrations on the permeate side were measured by using ICP–MS (ICAP Qc, ThermoFisher, Germany).

### Ion conductivity of BNC–GLP_100_

The ion conductivity (*κ*) of BNC–GLP_100_ can be defined as

2 $$\kappa=\frac{{I}_{d}}{{V}_{b}}\times \frac{L}{S}=G\times \frac{L}{S}$$

where *I*_d_ is the ion current measured at the applied bias (*V*_b_), *G* is the ion conductance, *S* is the cross-sectional area of the nanochannel, and *L* is the length of the PI nanochannel. Here, *S* is the effective area of the BNC–GLP. The radius profile *r*(*x*) for the conical nanochannel was calculated as follows^40^:

3 $$r(x)={d}_{{tip}}+\frac{({d}_{{base}}-{d}_{{tip}})}{L}x$$

Thus, the *L*/*S* of the conical nanochannel can be expressed as

4 $$\frac{L}{S}={\int }_{\!\!\!\! 0}^{L}\frac{1}{\pi {r}^{2}(x)}{dx}={\int }_{\!\!\!\! 0}^{L}\frac{1}{\pi {\left[{d}_{{tip}}+\frac{({d}_{{base}}-{d}_{{tip}})}{L}\right]}^{2}}{dx}$$

For a conical nanochannel, a simple approximation of *S*, namely, the geometric mean, can be adopted^41^:

5 $$S=\frac{4}{\pi {d}_{{base}}{d}_{{tip}}}$$

Consequently, the ion conductivity (*κ*) of BNC–GLP_100_ was calculated via the equation:

6 $$\kappa=G\frac{4L}{\pi {d}_{{base}}{d}_{{tip}}}$$

### Ion mobility in BNC–GLP_100_

To gain deep insights into the transport behaviors of Ln^3+^ ions in BNC–GLP_100_, the ion mobilities of these ions were measured by conducting drift-diffusion experiments at the applied voltages ranging from −0.4 to 0.4 V versus the Ag/AgCl electrode. Similar to the *I*–*V* measurements, the BNC–GLP_100_ membrane was clamped between two chambers. The cell facing the base of the conical nanochannel was filled with 100 mM of the chloride salt solution, while the other cell was filled with 10 mM of the same solution. The real zero-current voltage *E*_re_ (*E*_re_ = *E*_m_−*E*_redox_) was obtained from the measured potential (*E*_m_) by deducting the corresponding redox potential (*E*_redox_). The redox potential generated at the electrodes can be calculated as follows^42^:

7 $${E}_{{redox}}=\frac{{RT}}{{zF}}{{{{{\mathrm{ln}}}}}}\frac{{\alpha }_{H}{c}_{H}}{{\alpha }_{L}{c}_{L}}$$

where *R*, *T*, *z*, and *F* are the universal gas constant, temperature, ion valence, and Faraday’s constant, respectively. The *α* variables with subscripts *H* and *L* represent the activity coefficients at a high concentration *c*_H_ (100 mM) and low concentration *c*_L_ (10 mM), respectively. From these results, the mobility ratio of cations (*μ*_+_) to anions (*μ*_−_), *μ*_+_/*μ*_−_, was determined using the Henderson equation^43^:

8 $$\frac{{\mu }_{+}}{{\mu }_{-}}=-\frac{{z}_{+}}{{z}_{-}}\frac{{{{{{\mathrm{ln}}}}}}\Delta -{z}_{-}F{E}_{{re}}/{RT}}{{{{{{\mathrm{ln}}}}}}\Delta -{z}_{+}F{E}_{{re}}{RT}}$$

where *z* is to the valence of the cations and anions; *F* and *R* are the Faraday’s constant and universal gas constant, respectively; and *Δ* represents the concentration gradient of the electrolyte across the membrane (*Δ* = 10). We also measured the conductivities of various chloride solutions at a high concentration of 100 mM to neglect the surface charge contribution using the formula:

9 $$\kappa={10}^{3}({\mu }_{+}{c}_{+}-{\mu }_{-}{c}_{-}){N}_{A}e$$

where *c*_+_ and *c*_–_ are the concentrations of the anions and cations, respectively; *N*_A_ and *e* are the Avogadro constant and electron charge, respectively. Combining the Eqs. (8) and (9), the cation mobility of BNC–GLP_100_ was obtained.

### Effect of the BNC on Ln^3+^ ion transport

We also examined the effect of the BNC on Ln^3+^ ion transport to describe the differences between various Ln^3+^ ions and GLPs in the nanochannel. The ion current (*I*_d_) through BNC–GLP_100_ was calibrated by deducting the corresponding blank current (*I*_b_) measured by using the unmodified PI nanochannel. The obtained values of *I*_d_–*I*_b_ were normalized according to the formula of (*I*_d_–*I*_b_)/*I*_min_, where *I*_min_ is the ion current of Yb^3+^ ion.

### Theoretical calculations

All calculations in this study were performed by using the Gaussian 09 software package. Full geometry optimizations were performed to locate all stationary points and transition states (TSs) in water using the PBE0 method, def2TZVP basis set for C, H, and O atoms, and SDD (effective core potential) basis set for La, Nd, Eu, Td, Dy, and Yb atoms^44^. The self-consistent reaction field (SCRF) method based on the universal solvation model SMD was adopted to evaluate the solvent effect^45^. Dispersion corrections were computed by using the Grimme’s D3 (BJ) optimization method. Unless specified otherwise, the Gibbs free energies corrected for the solvation process were used in the discussion.

### Supplementary information

Supplementary Information

Peer Review File

## Data Availability

The data that support the findings of this study are available from the corresponding author upon request.

## References

[1] Cheisson T, Schelter EJ (2019). Rare earth elements: Mendeleev’s bane, modern marvels. Science.

[2] Bogart JA (2016). Accomplishing simple, solubility-based separations of rare earth elements with complexes bearing size-sensitive molecular apertures. Proc. Natl Acad. Sci. USA.

[3] Liu T, Chen J (2021). Extraction and separation of heavy rare earth elements: a review. Sep. Purif. Technol..

[4] Qiao Y, Schelter EJ (2018). Lanthanide photocatalysis. Acc. Chem. Res..

[5] Uda T, Jacob KT, Hirasawa M (2000). Technique for enhanced rare earth separation. Science.

[6] Yin X (2017). Rare earth separations by selective borate crystallization. Nat. Commun..

[7] Ni X-L (2015). Advances in the lanthanide metallosupramolecular chemistry of the cucurbit[n]urils. Coord. Chem. Rev..

[8] Minasian SG (2009). A comparison of 4*f vs* 5*f* metal-metal bonds in (CpSiMe_3_)_3_M-ECp* (M = Nd, U; E = Al, Ga; Cp* = C_5_Me_5_): synthesis, thermodynamics, magnetism, and electronic structure. J. Am. Chem. Soc..

[9] Chen L (2018). An overview on membrane strategies for rare earths extraction and separation. Sep. Purif. Technol..

[10] Bogart JA, Lippincott CA, Carroll PJ, Schelter EJ (2015). An operationally simple method for separating the rare-earth elements neodymium and dysprosium. Angew. Chem. Int. Ed..

[11] Goodwin CAP (2021). Structural and spectroscopic comparison of soft‐Se vs. hard‐O donor bonding in trivalent americium/neodymium molecules. Angew. Chem. Int. Ed..

[12] Higgins RF (2020). Magnetic field directed rare-earth separations. Angew. Chem. Int. Ed..

[13] Ashour RM (2017). Selective separation of rare earth ions from aqueous solution using functionalized magnetite nanoparticles: kinetic and thermodynamic studies. Chem. Eng. J..

[14] Park DM (2016). Bioadsorption of rare earth elements through cell surface display of lanthanide binding tags. Environ. Sci. Technol..

[15] Simonnet M, Kobayashi T, Shimojo K, Yokoyama K, Yaita T (2021). Study on phenanthroline carboxamide for lanthanide separation: Influence of amide substituents. Inorg. Chem..

[16] Cotruvo JA, Featherston ER, Mattocks JA, Ho JV, Laremore TN (2018). Lanmodulin: a highly selective lanthanide-binding protein from a lanthanide-utilizing bacterium. J. Am. Chem. Soc..

[17] Dong Z (2021). Bridging hydrometallurgy and biochemistry: a protein-based process for recovery and separation of rare earth elements. ACS Cent. Sci..

[18] Brigham DM (2017). Trefoil-shaped outer-sphere ion clusters mediate lanthanide(III) ion transport with diglycolamide ligands. J. Am. Chem. Soc..

[19] Slope LN, Daubney OJ, Campbell H, White SA, Peacock AFA (2021). Location-dependent lanthanide selectivity engineered into structurally characterized designed coiled coils. Angew. Chem. Int. Ed..

[20] Liu Y, Zhu L, Sun X, Chen J (2010). Toward greener separations of rare earths: Bifunctional ionic liquid extractants in biodiesel. AIChE J..

[21] Meng C (2022). Angstrom-confined catalytic water purification within Co-TiO_*x*_ laminar membrane nanochannels. Nat. Commun..

[22] Doyle DA (1998). The structure of the potassium channel: molecular basis of K^+^ conduction and selectivity. Science.

[23] Li X (2022). Microorganisms accelerate REE mineralization in supergene environments. Appl. Environ. Microbiol..

[24] Featherston ER, Issertell EJ, Cotruvo JA (2021). Probing lanmodulin’s lanthanide recognition via sensitized luminescence yields a platform for quantification of terbium in acid mine drainage. J. Am. Chem. Soc..

[25] Mattocks JA, Ho JV, Cotruvo JA (2019). A selective, protein-based fluorescent sensor with picomolar affinity for rare earth elements. J. Am. Chem. Soc..

[26] Deblonde GJ (2020). Selective and efficient biomacromolecular extraction of rare-earth elements using lanmodulin. Inorg. Chem..

[27] Xin W (2022). Biomimetic KcsA channels with ultra-selective K^+^ transport for monovalent ion sieving. Nat. Commun..

[28] Liu Q (2015). Engineered ionic gates for ion conduction based on sodium and potassium activated nanochannels. J. Am. Chem. Soc..

[29] Gohil H, Chatterjee S, Yadav S, Suresh E, Paital AR (2019). An ionophore for high lithium loading and selective capture from brine. Inorg. Chem..

[30] Pintacuda G, John M, Su X-C, Otting G (2007). NMR structure determination of protein-ligand complexes by lanthanide labeling. Acc. Chem. Res..

[31] Bertini I, Janik MBL, Lee Y-M, Luchinat C, Rosato A (2001). Magnetic susceptibility tensor anisotropies for a lanthanide ion series in a fixed protein matrix. J. Am. Chem. Soc..

[32] Sherry AD, Singh M, Geraldes CFGC (1986). Nuclear magnetic resonance structural studies of an axially symmetric lanthanide ion chelate in aqueous solution. J. Magn. Reson..

[33] Funke H, Scheinost AC, Chukalina M (2005). Wavelet analysis of extended x-ray absorption fine structure data. Phys. Rev. B.

[34] Wang G (2023). A green and efficient technology to recover rare earth elements from weathering crusts. Nat. Sustain..

[35] Tasaki-Handa Y (2016). Separation of neodymium and dysprosium by forming coordination polymers. Sep. Purif. Technol..

[36] Jha MK (2016). Review on hydrometallurgical recovery of rare earth metals. Hydrometallurgy.

[37] Chen L, Chen J (2016). Asymmetric membrane containing ionic liquid [A336][P507] for the preconcentration and separation of heavy rare earth lutetium. ACS Sustain. Chem. Eng..

[38] Geraldes CFGC, Alpoim MC, Marques MPM, Sherry AD, Singh M (1985). Nuclear magnetic resonance and potentiometric studies of the protonation scheme of a triaza triacetic macrocycle and its complexes with lanthanum and lutetium. Inorg. Chem..

[39] Bauer, D. et al. U.S. Department of Energy, Critical Materials Strategy Summary, Chapter 2. https://www.energy.gov/sites/prod/files/edg/news/documents/criticalmaterialsstrategy.pdf (2010).

[40] Siwy Z, Fuliński A (2002). Fabrication of a synthetic nanopore ion pump. Phys. Rev. Lett..

[41] Apel PY, Korchev YE, Siwy Z, Spohr R, Yoshida M (2001). Diode-like single-ion track membrane prepared by electro-stopping. Nucl. Instrum. Methods Phys. Res. Sect. B.

[42] Tunuguntla RH (2017). Enhanced water permeability and tunable ion selectivity in subnanometer carbon nanotube porins. Science.

[43] Perram JW, Stiles PJ (2006). On the nature of liquid junction and membrane potentials. Phys. Chem. Chem. Phys..

[44] Krishnan R, Binkley JS, Seeger R, Pople JA (1980). Self‐consistent molecular orbital methods. XX. A basis set for correlated wave functions. J. Chem. Phys..

[45] Marenich AV, Cramer CJ, Truhlar DG (2009). Performance of SM6, SM8, and SMD on the SAMPL1 test set for the prediction of small-molecule solvation free energies. J. Phys. Chem. B.

