# Supplementary material for: High-efficiency dysprosium-ion extraction enabled by a biomimetic nanofluidic channel
Source: Nat Commun. 2024 Jul 12;15:5876. doi: 10.1038/s41467-024-50237-9 (PMC11245470; doi:10.1038/s41467-024-50237-9)
Supplement: Supplementary file 1 — Supplementary Information [file 41467_2024_50237_MOESM1_ESM.pdf]

Supplementary Information

Weiwen Xin[1], Yanglansen Cui[1], Yongchao Qian[1], Tianchi Liu[1], Xiang-Yu Kong[1,2,3,4] ✉, Haoyang Ling[1,2], Weipeng Chen[1], Zhehua Zhang[1,2], Yuhao Hu[1], Lei Jiang[1,2,3,4], and Liping Wen[1,2,3,4,5] ✉

[1] CAS Key Laboratory of Bio-inspired Materials and Interfacial Science, Technical Institute of Physics and Chemistry, Chinese Academy of Sciences, 100190 Beijing, PR China.

[2] School of Future Technology, University of Chinese Academy of Sciences, 100049 Beijing, PR China.

[3] School of Chemistry and Materials Science, University of Science and Technology of China, Hefei, 230026 Anhui, PR China.

[4] Suzhou Institute for Advanced Research, University of science and Technology of China, Suzhou, 215123 Jiangsu, PR China.

[5] Qingdao Institute of Bioenergy and Bioprocess Technology, Chinese Academy of Sciences, 266101 Qingdao, PR China.

✉ e-mail: wen@mail.ipc.ac.cn; kongxiangyu@mail.ipc.ac.cn

This file includes:

Supplementary Methods

Supplementary Figures 1 to 24

Supplementary Tables 1 to 13

Supplementary References

Table of Contents

1. Supplementary Methods

1.1. Chemicals.

1.2. X-ray photoelectron spectroscopy (XPS) measurements.

1.3. Scanning electron microscopy (SEM).

1.4. Inductively coupled plasma mass spectrometry (ICP-MS).

1.5. X-ray diffraction (XRD) measurements.

1.6. Neodymium-dysprosium solutions.

1.7. Uncertainty analysis.

2. Supplementary Figures

Supplementary Figure 1. Fabrication of biomimetic nanofluidic channel.

Supplementary Figure 2. SEM images of nanochannel.

Supplementary Figure 3. Characteristics of BNC−GLP_{150}.

Supplementary Figure 4. Stability of GLP in different conditions for a long-term use.

Supplementary Figure 5. Effect of BNC on ion transport determined by the changes of ion current.

Supplementary Figure 6. Partial ^{1}H NMR analyses.

Supplementary Figure 7. Raw ITC thermograms.

Supplementary Figure 8. Variations in the observed heat changes plotted against the molar ratios of Ln^{3+} ions to GLP determined by titrating the GLP solutions with Ln^{3+} solutions.

Supplementary Figure 9. XRD patterns of the composites of GLP and $LnCl_3$.

Supplementary Figure 10. XPS measurements of GLP after binding Ln^{3+} ions.

Supplementary Figure 11. Analysis of La L_3-edge XANES spectra.

Supplementary Figure 12. Analysis of Eu L_3-edge XANES spectra.

Supplementary Figure 13. Analysis of Tb L_3-edge XANES spectra.

Supplementary Figure 14. Analysis of Yb L_3-edge XANES spectra.

Supplementary Figure 15. Analysis of Dy and Nd L_3-edge XANES spectra.

Supplementary Figure 16. Analysis of Dy and Nd L_3-edge XANES spectra.

Supplementary Figure 17. WT-EXAFS Ln L_3-edge spectra of Ln-GLP and their references.

Supplementary Figure 18. Setup of ion permeation through BNC−GLP_{100}.

Supplementary Figure 19. Comparison of ion concentration measured by ICP-MS measurements.

Supplementary Figure 20. Ion concentration of Dy^{3+} and Nd^{3+} in the binary solution.

Supplementary Figure 21. The change of ion concentration through BNC−GLP_{100}.

Supplementary Figure 22. Cycle of Dy^{3+} extraction by using the biomimetic nanochannel and the corresponding percentage contents of consecutive cycles.

Supplementary Figure 23. Transport rates of other heavy Ln^{3+} ions.

Supplementary Figure 24. Theoretical calculations for visualizing the interactions between heavy Ln^{3+} ions and GLP.

3. Supplementary Tables

Supplementary Table 1. The differences of Ln^{3+} with different coordination numbers on their ion radii.

Supplementary Table 2. The La series divided into light REEs (LREEs) and heavy REEs (HREEs).

Supplementary Table 3. The conduction of Ln^{3+} ions in the bulk.

Supplementary Table 4. Structure of electronic shell of Ln^{3+} ions.

Supplementary Table 5. EXAFS fitting parameters of Dy_2O_3, Dy-GLP, Nd_2O_3, and Nd-GLP.

Supplementary Table 6. EXAFS fitting parameters of La_2O_3, La-GLP, Eu_2O_3, and Eu-GLP, Tb_2O_3, Tb-GLP, Yb_2O_3, and Yb-GLP.

Supplementary Table 7. Atomistic coordinates for the mode of the La^{3+}-GLP compound optimized by using the PBE0 method.

Supplementary Table 8. Atomistic coordinates for the mode of the Nd^{3+}-GLP compound optimized by using the PBE0 method.

Supplementary Table 9. Atomistic coordinates for the mode of the Eu^{3+}-GLP compound optimized by using the PBE0 method.

Supplementary Table 10. Atomistic coordinates for the mode of the Tb^{3+}-GLP compound optimized by using the PBE0 method.

Supplementary Table 11. Atomistic coordinates for the mode of the Dy^{3+}-GLP compound optimized by using the PBE0 method.

Supplementary Table 12. Atomistic coordinates for the mode of the Yb^{3+}-GLP compound optimized by using the PBE0 method.

Supplementary Table 13. Parameters (K_a, n, ΔH_a, $T\Delta S_a$, ΔG_a) of ITC measurements for different REE ions.

4. Supplementary References

1. Supplementary Methods

1.1. Chemicals.

Glycyl-L-proline (GLP, >99.5%) was purchased from Sigma-Aldrich. Lanthanide chlorides, including $LaCl_3$ (99.99%), $NdCl_3$ (99.99%), $EuCl_3$ (99.99%), $TbCl_3$ (99.99%), $DyCl_3$ (99.99%), $YbCl_3$ (99.99%), La_2O_3 (99.99%), Nd_2O_3 (99.99%), Eu_2O_3 (99.99%), Tb_2O_3 (99.99%), Dy_2O_3 (99.99%), $HoCl_3$ (99.99%), $ErCl_3$ (99.99%), $TmCl_3$ (99.99%) and Yb_2O_3 (99.99%) were obtained from Beijing Innochem Science & Technology co., LTD. Lithium chloride (LiCl, 99.99%), sodium chloride (NaCl, 99.99%), potassium chloride (KCl, 99.99%), magnesium chloride ($MgCl_2$, 99.99%), and calcium chloride ($CaCl_2$, 99.99%) were purchased from *J&K* Beijing Co., Ltd. 1-ethyl-3-(3-dimethyllaminopropyl) carbodiimide (EDC, >99%), N-hydroxysuccinimide (NHSS, >99%), 14 wt.% sodium hypochlorite (NaClO, >99%), and potassium iodide (KI, 99.99%) were purchased from Sigma-Aldrich. The polyimide (PI) membranes employed in this work are based on an UMAT experiment, performed at the beam line X0 at the GSI Helmholtzzentrum für Schwerionenforschung, Darmstadt (Germany) in the frame of FAIR Phase 0. All chemicals were used without further purification. All the experimental solutions were prepared using degassed Milli-Q water (18.2 MΩ cm^{-1}).

1.2. X-ray photoelectron spectroscopy (XPS) measurements.

XPS spectra were performed in an ultrahigh-vacuum system with a base pressure of less than 5×10^{-10} mbar using an Al Ka source (ESCALAB 250Xi) and a power of 300 W. To confirm the strong interactions between GLP and metal ions, these samples were modified onto the surface of the PI membrane, which could be monitored directly. This method has been widely used for obtaining the information of ions in nanochannels.

Figure 1g shows the K analysis of the etched PI surface (blue lines) and the GLP-modified PI surface (green, orange, and red lines). The considerable decreases of the K peak prove that GLP molecules are grafted successfully, and there are still residual carboxyl groups on the modified PI surface when the GLP concentrations were 25 and 50 mM. In detail, lots of

carboxyl groups (COO^-) were obtained on the surfaces of the etched PI nanochannel. Then K^+ ions were added to combine with COO^- where the K signal can be detected by XPS measurement. After that, a small number of GLPs such as 25 and 50 mM were employed to replace K^+ ions owing to the strong covalent interactions between GLP and COO^-. In that case, the K signal decreased, indicating some GLPs were successfully grafted. In the right amount of GLP, all of K^+ ions were replaced by GLPs, for example the concentration of GLP is 100 mM. The final coverage of GLP is estimated to be ~100% when GLP concentrations were 100 and 150 mM (Supplementary Fig. 3), and the average density of carboxylate groups on the etched PI surface has been estimated to be ~1.5 groups nm^{-2}.

1.3. Scanning electron microscopy (SEM).

Field-emission SEM (Hitachi S-4800 scanning electron microscope with an accelerating voltage of 10 kV coupled with second electron imaging was performed to observed the structural details of the nanochannel. In order to obtain the high-quality image information, the Au nanolayer was deposited onto the surface of the nanochannel by ion sputtering with an Au target (99.999%) using an ion sputtering system (SBC-12, KYKY Technology Development Ltd.) in a vacuum (>4.0 MPa) for 60 seconds.

1.4. Inductively coupled plasma mass spectrometry (ICP-MS).

Inductively coupled plasma mass spectrometry (ICP-MS) was employed to determine metal-ion concentrations. Every sample was tested at least three times to observe the average values.

1.5. X-ray diffraction (XRD) measurements.

X-ray diffraction (XRD) measurements were accomplished on Micromeritics Tristar II 3020 with a PANalytical B.V. Empyrean powder diffractometer using Cu-Kα radiation at 40 kV and 40 mA over a range of 2θ = 4.0° up to 40.0° with a step size of 0.02° and 2s per step.

1.6. Neodymium-dysprosium solutions.

Neodymium (Nd) is an important component of sintered neodymium magnets. Dysprosium (Dy) are also the key components of this material, which increase its intrinsic coercivity. High-performance Nd-magnets include up to 9% Dy by total magnet weight. The U.S. Department of Energy has categorized dysprosium and neodymium as critical materials because of their supply problems and importance to technology. In this work, we prepared high-concentration Nd solutions with low-concentration Dy (Nd/Dy = 32.3:1, wt.%).

1.7. Uncertainty analysis.

In this work, uncertainty originates mainly from two routes: experiments (Uncertainty Type A, μ_A) and measurements (Uncertainty Type B, μ_B). For accuracy, each experiment was repeated at least three times, and the μ_A was calculated by equation:

$$\mu_A = \sqrt{\frac{1}{n}\sum_{i=1}^{n}(x_i - \bar{x})^2}$$

where n is the experimental repetition time, x_i is the result of experiment i, and $\bar{x}$ is the average result of n experiments which can be calculated by the equation:

$$\bar{x} = \frac{1}{n}\sum_{i=1}^{n} x_i$$

For ITC measurement, the analytical precision of the instrument (TA NANO) was better than 5% relative standard deviation, and the Type B uncertainty (μ_B-ITC) of an experiment (*x*) was calculated to be:

$$\mu_{B-ITC} = \frac{5\%x}{\sqrt{3}}$$

Additionally, the qualities of various chemicals were weighed using an analytical balance (Mettler Toledo) with the analytical precision of ±0.1 mg and thus, the uncertainty was calculated to be:

$$\mu_{B-Qual} = \frac{0.1}{\sqrt{3}}$$

The pH calibration was performed using FE30 (Mettler Toledo) with the analytical precision of ±0.01, and the corresponding uncertainty was calculated to be:

$$\mu_{B-pH} = \frac{0.01}{\sqrt{3}}$$

Finally, the combined standard uncertainty (μ) was calculated by equation:

$$\mu = \sqrt{\mu_A^2 + \mu_B^2}$$

2. Supplementary Figures

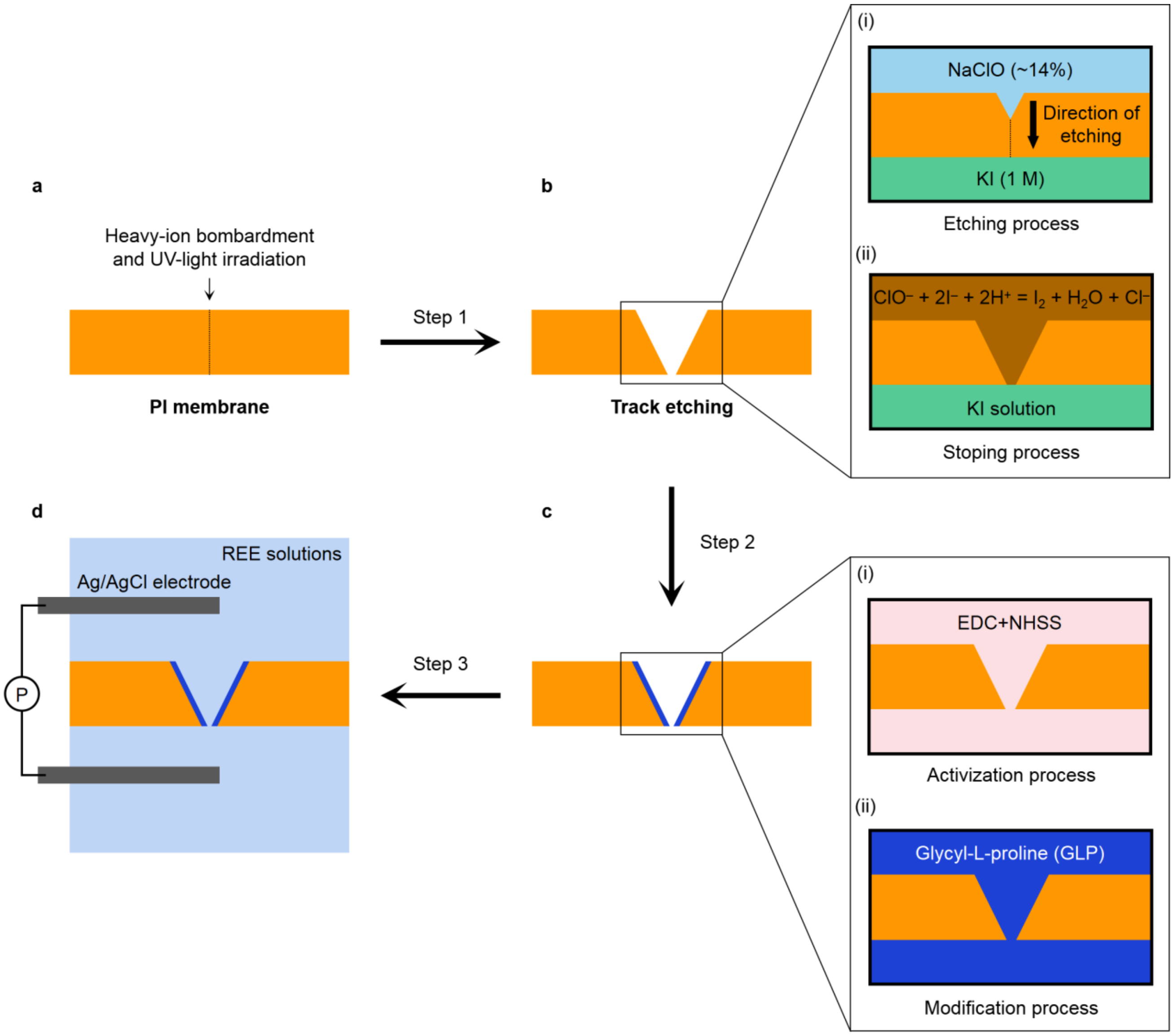

Supplementary Figure 1. Fabrication of biomimetic nanofluidic channel. The process was divided into three steps, including 1. Irradiation of the PI membrane using UV light; 2. Ion-track etching using NaClO solutions under an applied bias to obtain the single nanochannel; and 3. Modification of GLP into PI nanochannel. In detail, (a) Two faces of polyimide (PI) membrane were first irradiated with an ultraviolet (UV) light for 1 h. (b) The single conical nanochannel was obtained by using the well-developed etching technique at 333 K and a bias of +1 V. The etching process was stopped at a desired current value corresponding to a certain tip diameter. For more details, the PI membrane was clamped between two polytetrafluoroethylene (PTFE) compartments, of which one cell, facing the base of the conical nanochannel, was filled with ~14 wt.% NaClO solution as the etching solutions, while

the other cell was filled with 1 M KI solution in order to neutralize the etchant as soon as the channel opened. (c) The carboxyl group surface was activated by soaking in EDC/NHSS aqueous solution for 1 h at 298 K without light. Once finished, the PI membrane was immersed into GLP solutions with different concentrations, such as, 25, 50, 100, and 150 mM. Note that the tip of channel was in contact with GLP solutions. Resultant membrane was washed for several times using DI water. (d) The as-prepared BNC−GLP was clamped between two compartments, which was monitored by a Keithley 6487 picoammeter using the Ag/AgCl electrodes.

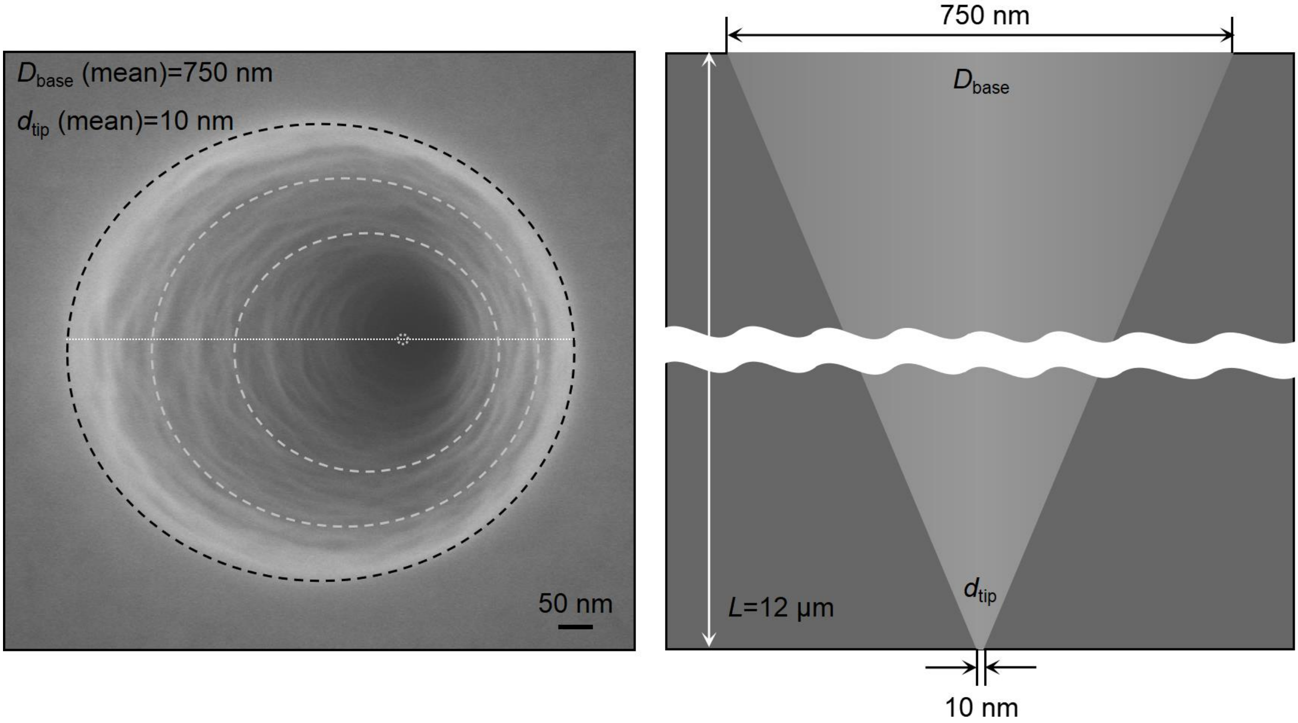

Supplementary Figure 2. SEM images of nanochannel. As a typical example of showing the dimension of the conical channel, we can calculate the diameter of the base to be 750 nm. The diameter of the tip can be found to be ~10 nm, which is described by the equation (Supplementary Equation 1):

$$d_{tip}=\frac{4lI}{\pi \kappa_c UD} \quad \text{(Supplementary Equation 1)}$$

where κ_c is the specific conductivity in 1 M KCl solution at 298 K, that is, 0.11173 Ω^{-1} cm^{-1}. I, U, l, and D refer to the ionic current through the nanochannel, the voltage, the length of the nanochannel, and the diameter of the base. In principle, the practical diameter of the modified nanochannel can be obtained according to the tip diameter of the unmodified nanochannel, which is equal to the unmodified diameter minus the two lengths of the modified GLP layer. In case of a GLP monolayer, the size of the modified nanochannel can be calculated to be approximate 8.5 nm.

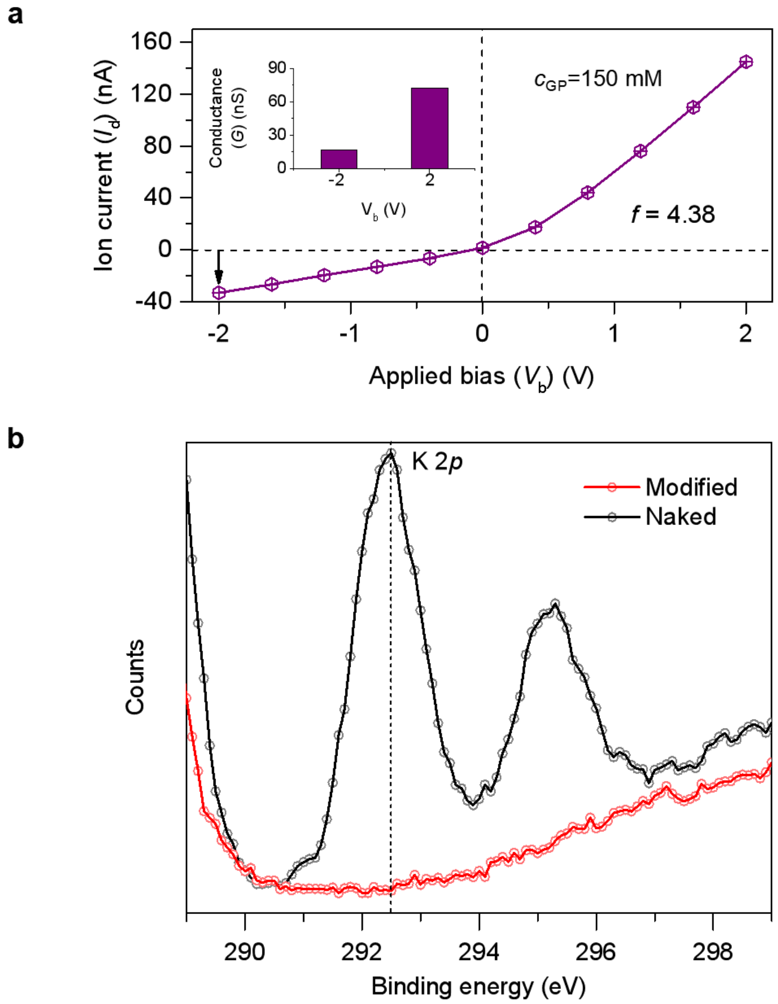

Supplementary Figure 3. Characteristics of BNC−GLP150. (a) I−V curve of the asymmetric ion channel functionalized by GLP with a concentration of 150 mM. In this case, the calculated ion rectification is 4.38. Due to the partial self-association of GLP, the carboxyl groups were shielded, which decreasing the ion conduction and rectification. (b) K 2p XPS spectra of the functionalized PI by 150 mM GLP.

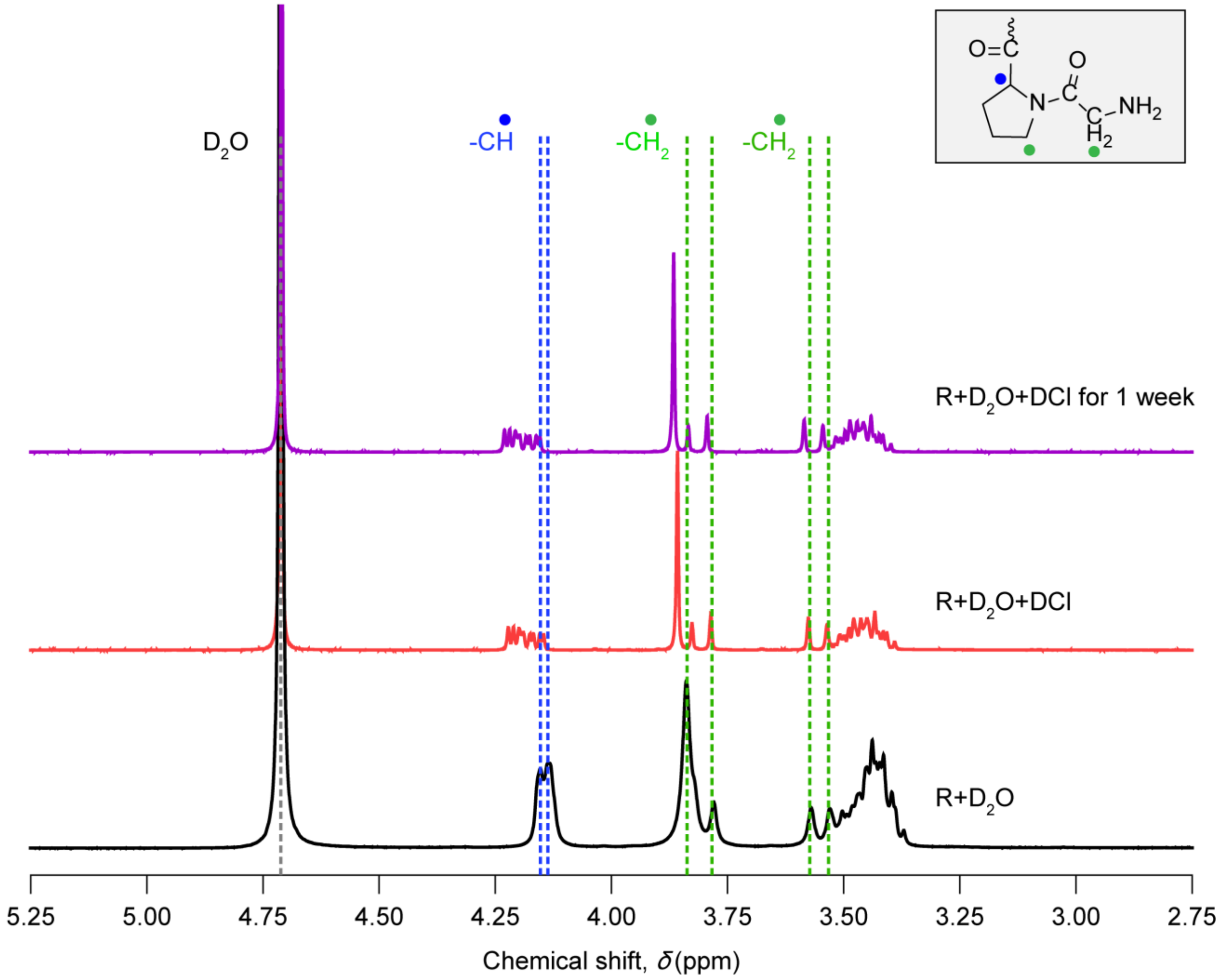

Supplementary Figure 4. Stability of GLP in different conditions for a long-term use. GLP (R) showed the excellent long-term stability in D_2O (black line), D_2O/DCl (pH=4, red line), and D_2O/DCl for one week (pH=4, purple line). Compared with the peaks of GLP in D_2O, the corresponding peaks of D_2O/DCl exhibited the few shifts, indicating GLP could be stably used.

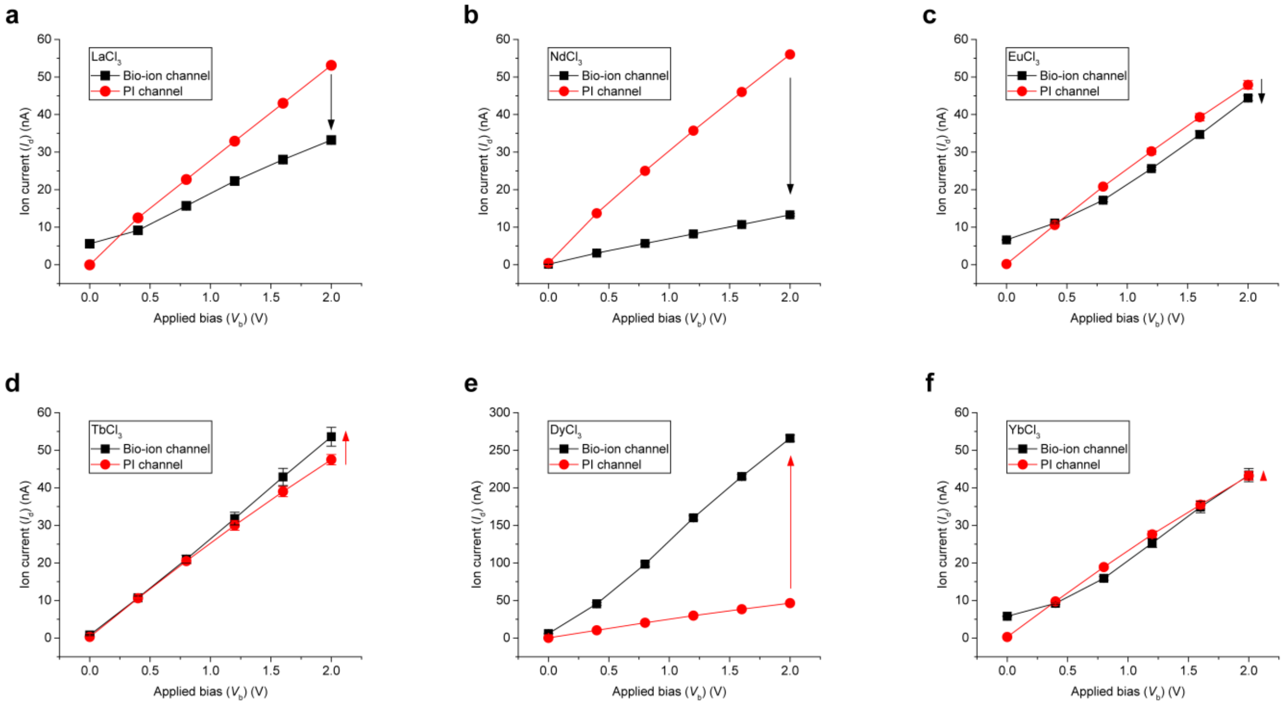

Supplementary Figure 5. Effect of BNC on ion transport determined by the changes of ion current. For a clear comparison, we measured the ion current of the unmodified PI nanochannel (red spots/lines) and the modified PI nanochannel (black spots/lines), that is BNC−GLP$_{100}$, respectively. As shown in Supplementary Figure 5, the ion currents of La^{3+} (a), Nd^{3+} (b), and Eu^{3+} (c) ions through the unmodified PI nanochannel are higher than those through the modified nanochannel, which suggesting that the BNC−GLP$_{100}$ suppresses their transport due to the weaker interactions between ions and surface of nanochannel. On the contrary, the ion currents of Tb^{3+} (d), Dy^{3+} (e), and Yb^{3+} (f) ions through the unmodified PI nanochannel are lower than those through the modified nanochannel, which suggesting that the BNC−GLP$_{100}$ enhances their transport due to the stronger interactions between ions and surface of nanochannel. Error bars give the standard deviation from three independent tests.

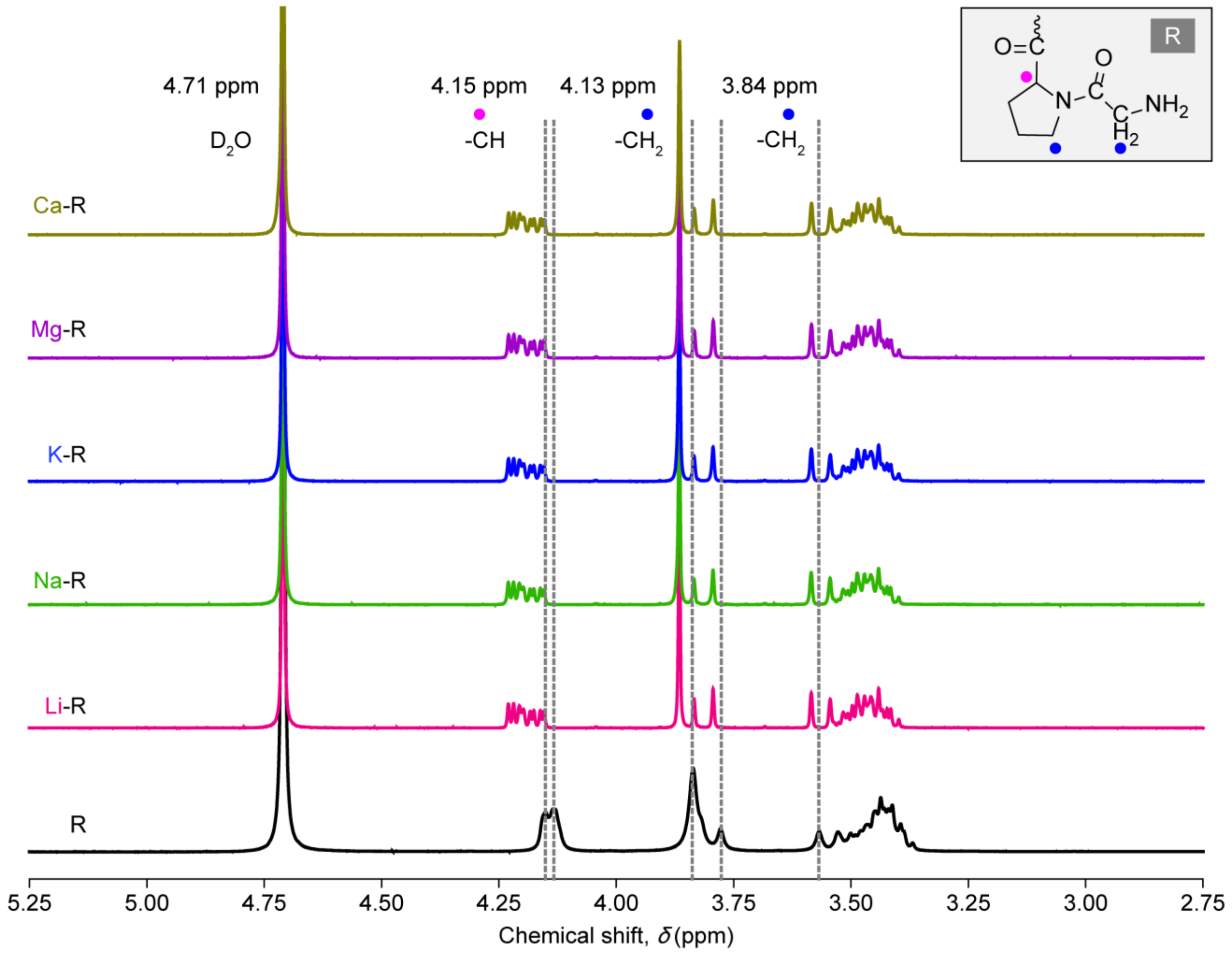

Supplementary Figure 6. Partial ^{1}H NMR analyses. ^{1}H NMR of GLP showed the proton resonance shifts for different REEs in D_2O (4.71 ppm), with TMS as an internal standard set at 0 ppm. The addition of REEs led to different shifts with respect to the reference peak (GLP, black line), with the extent of the shift indicating the corresponding interactions. The concentration of GLP and REEs was respectively set to 2 mM and 0.2 mM in D_2O. Inset shows the structure of GLP with H signals of $-CH_2$ at 3.84 and 4.13 ppm (blue spots) and −CH at 4.15 ppm (pink spots). ^{1}H chemical shifts were displayed in different colors according to specific species.

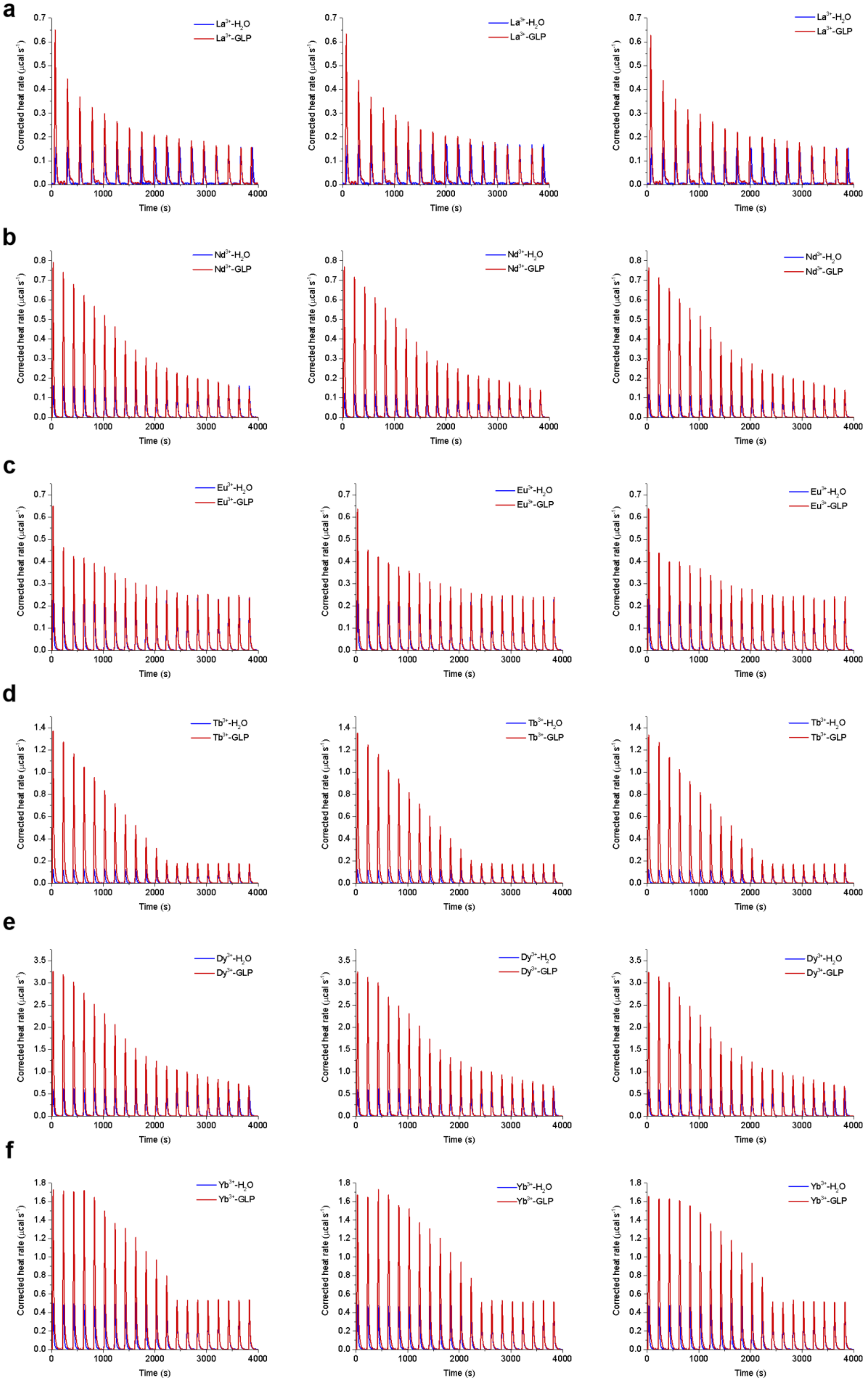
a
La3+-H2O
La3+-GLP
b
Nd3+-H2O
Nd3+-GLP
c
Eu3+-H2O
Eu3+-GLP
d
Tb3+-H2O
Tb3+-GLP
e
Dy3+-H2O
Dy3+-GLP
f
Yb3+-H2O
Yb3+-GLP
Corrected heat rate (μcal s-1)
Time (s)

Supplementary Figure 7. Raw ITC thermograms replicated for three times. (a) La^{3+}, (b) Nd^{3+}, (c) Eu^{3+}, (d) $Tb^{3+,}$ (e) Dy^{3+}, and (f) Yb^{3+}. Blue curves represent the results from control experiments of titrating various Ln^{3+} ion solutions into H_2O, and red curves represent the process by which various Ln^{3+} ion solutions are titrated into GLP solution. For all measurements, the pH value of solutions is fixed at 4.

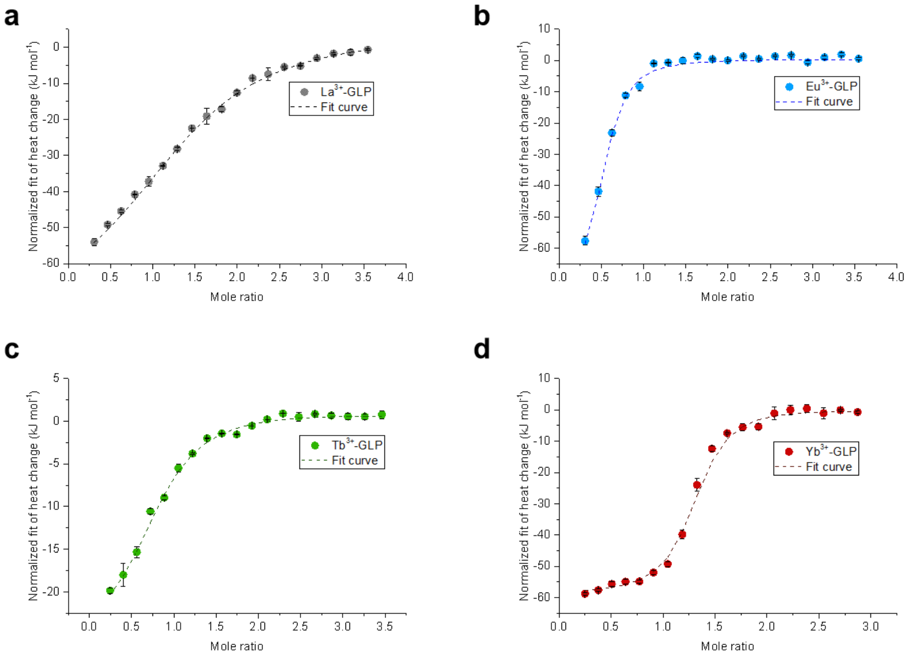

Supplementary Figure 8. Variations in the observed heat changes plotted against the molar ratios of Ln^{3+} ions to GLP determined by titrating the GLP solutions with Ln^{3+} solutions. (a) La^{3+}, (b) Eu^{3+}, (c) Tb^{3+}, and (d) Yb^{3+}. Error bars give the standard deviation from three independent tests.

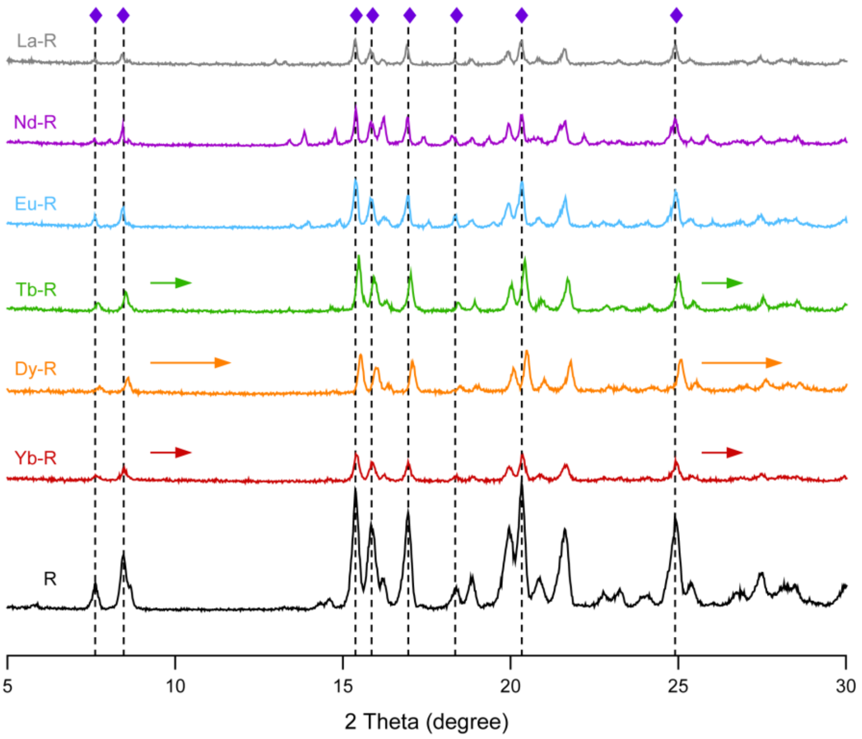

Supplementary Figure 9. XRD patterns of the composites of GLP and $LnCl_3$.

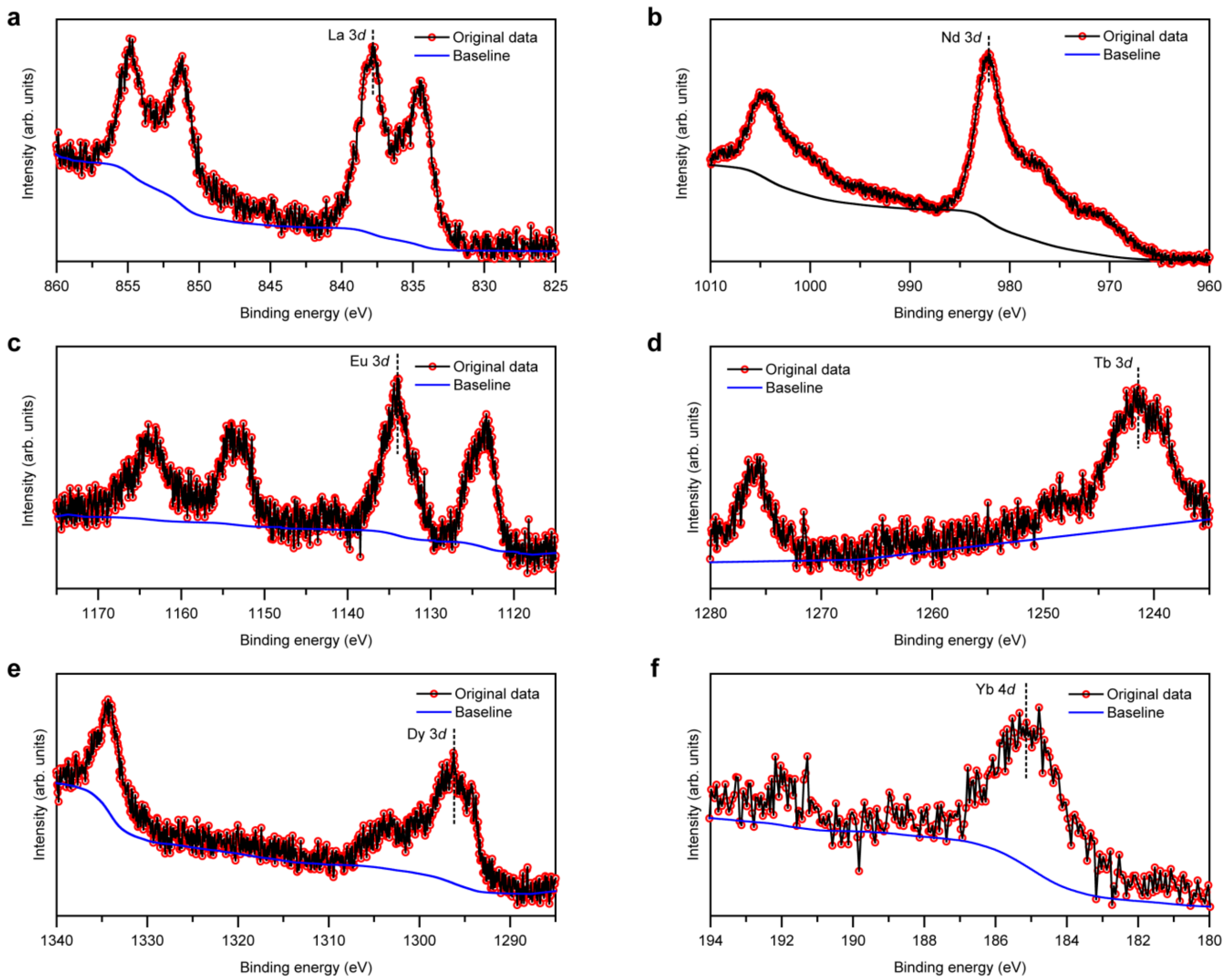

Supplementary Figure 10. XPS measurements of GLP after binding Ln^{3+} ions. These typical peaks were observed, including La 3*d* (a), Nd 3*d* (b), Eu 3*d* (c), Tb 3*d* (d), Dy 3*d* (e), and Yb 4*d* (f), providing evidence that GLP could interact with Ln^{3+} ions.

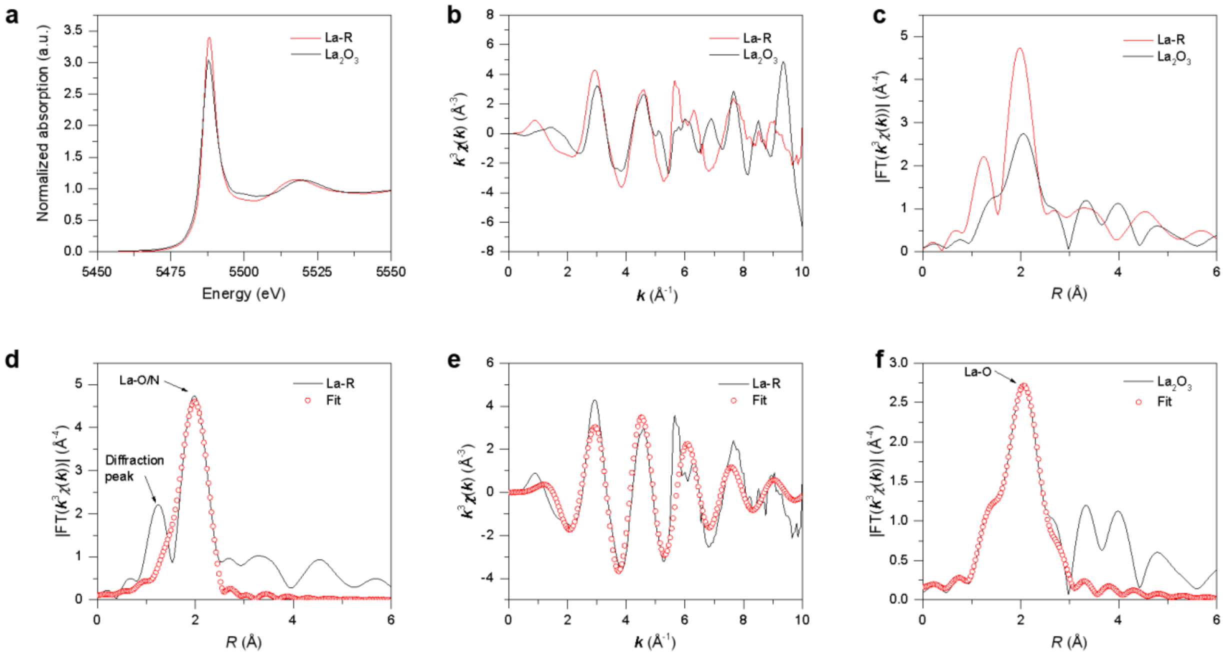

Supplementary Figure 11. Analysis of La L_3-edge XANES spectra. (a,d) Normalized L_3-edge XANES spectra of La_2O_3 and La-GLP. (b,e) k^3-weighted spectra in *k* space for La-GLP and La_2O_3. Measured and fitted spectra are matched very well for both samples. (c,f) Fourier-transform (FT)-EXAFS spectra fittings for La-GLP (e) and La_2O_3 (f) at La L_3-edge. R (Å) is the peak location scale for the Fourier-transformed magnitude in the real space.

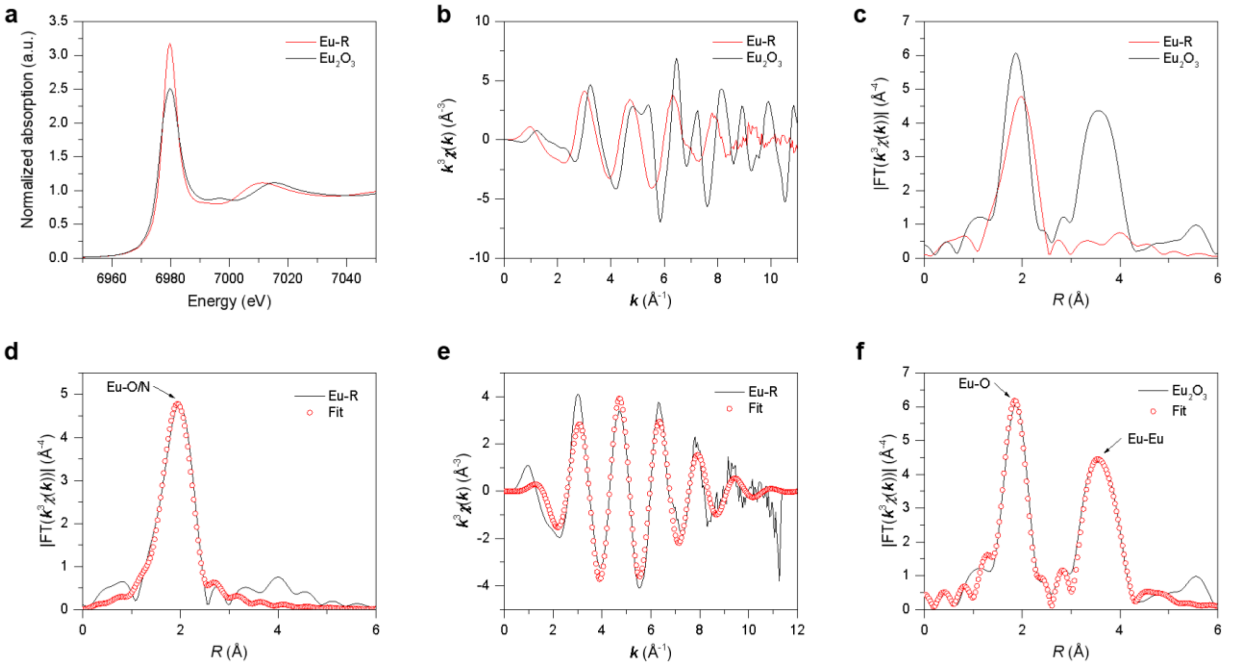

Supplementary Figure 12. Analysis of Eu L_3-edge XANES spectra. (a,d) Normalized L_3-edge XANES spectra of Eu_2O_3 and Eu-GLP. (b,e) k^3-weighted spectra in *k* space for Eu-GLP and Eu_2O_3. Measured and fitted spectra are matched very well for both samples. (c,f) Fourier-transform (FT)-EXAFS spectra fittings for Eu-GLP (e) and Eu_2O_3 (f) at Eu L_3-edge. R (Å) is the peak location scale for the Fourier-transformed magnitude in the real space.

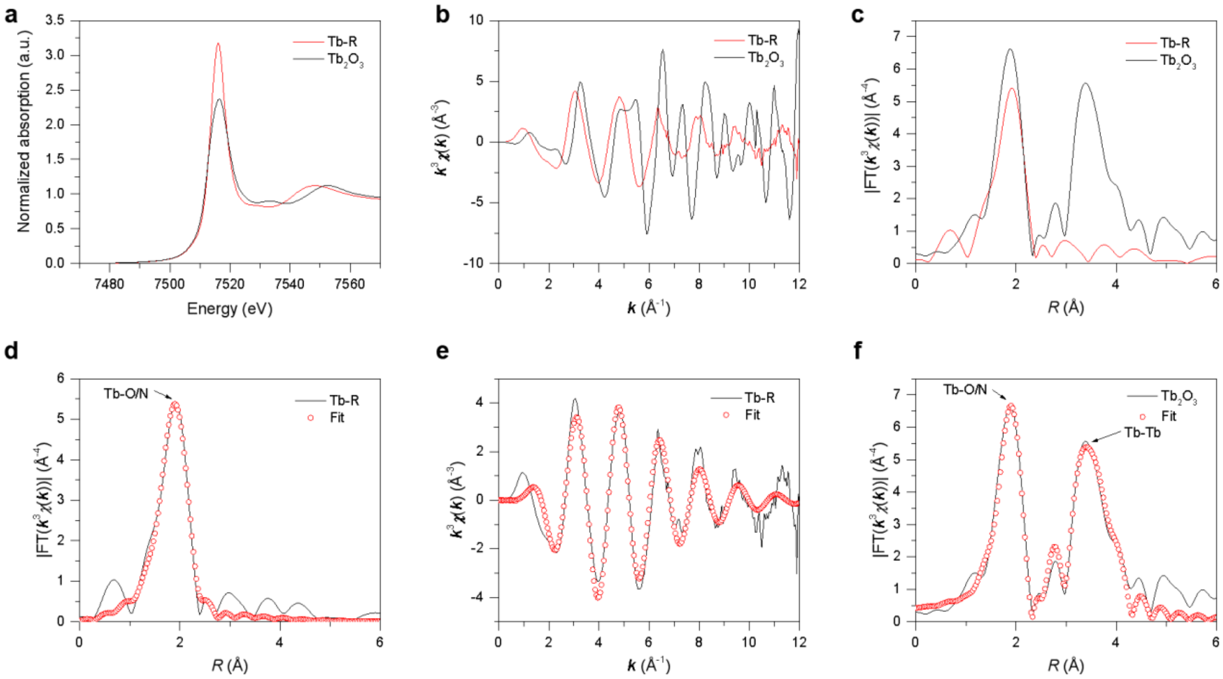

Supplementary Figure 13. Analysis of Tb L_3-edge XANES spectra. (a,d) Normalized L_3-edge XANES spectra of Tb_2O_3 and Tb-GLP. (b,e) k^3-weighted spectra in *k* space for Tb-GLP and Tb_2O_3. Measured and fitted spectra are matched very well for both samples. (c,f) Fourier-transform (FT)-EXAFS spectra fittings for Tb-GLP (e) and Tb_2O_3 (f) at Tb L_3-edge. R (Å) is the peak location scale for the Fourier-transformed magnitude in the real space.

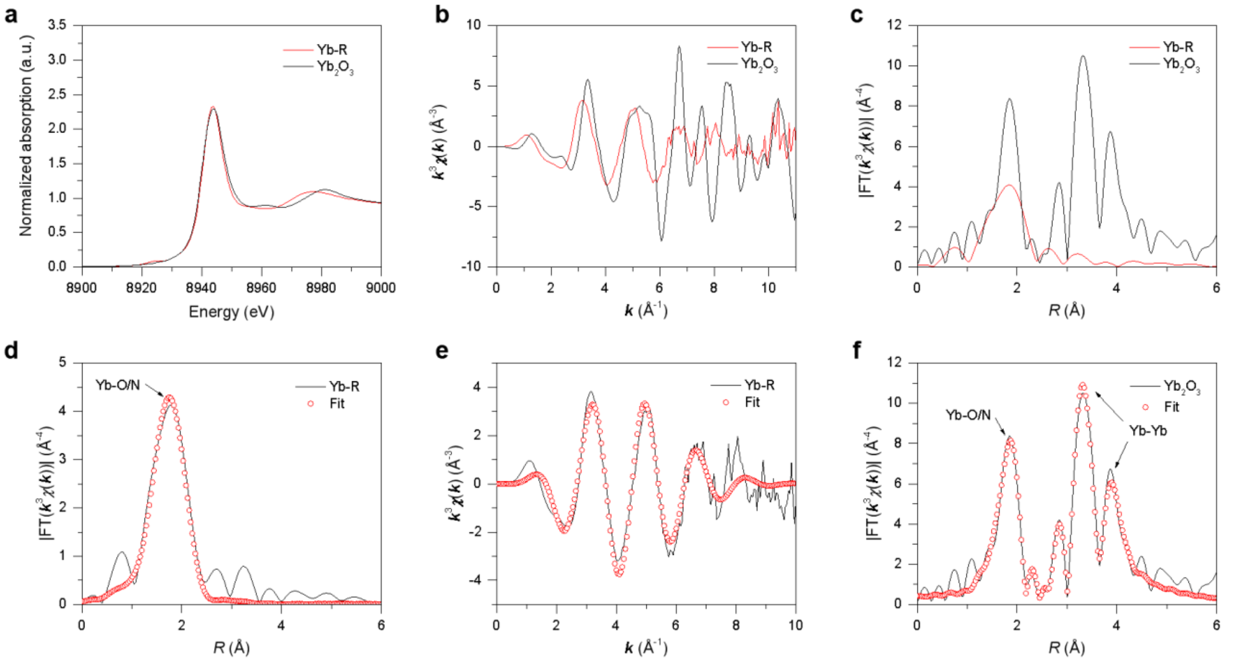

Supplementary Figure 14. Analysis of Yb L_3-edge XANES spectra. (a,d) Normalized L_3-edge XANES spectra of Yb_2O_3 and Yb-GLP. (b,e) k^3-weighted spectra in *k* space for Yb-GLP and Yb_2O_3. Measured and fitted spectra are matched very well for both samples. (c,f) Fourier-transform (FT)-EXAFS spectra fittings for Yb-GLP (e) and Yb_2O_3 (f) at Yb L_3-edge. R (Å) is the peak location scale for the Fourier-transformed magnitude in the real space.

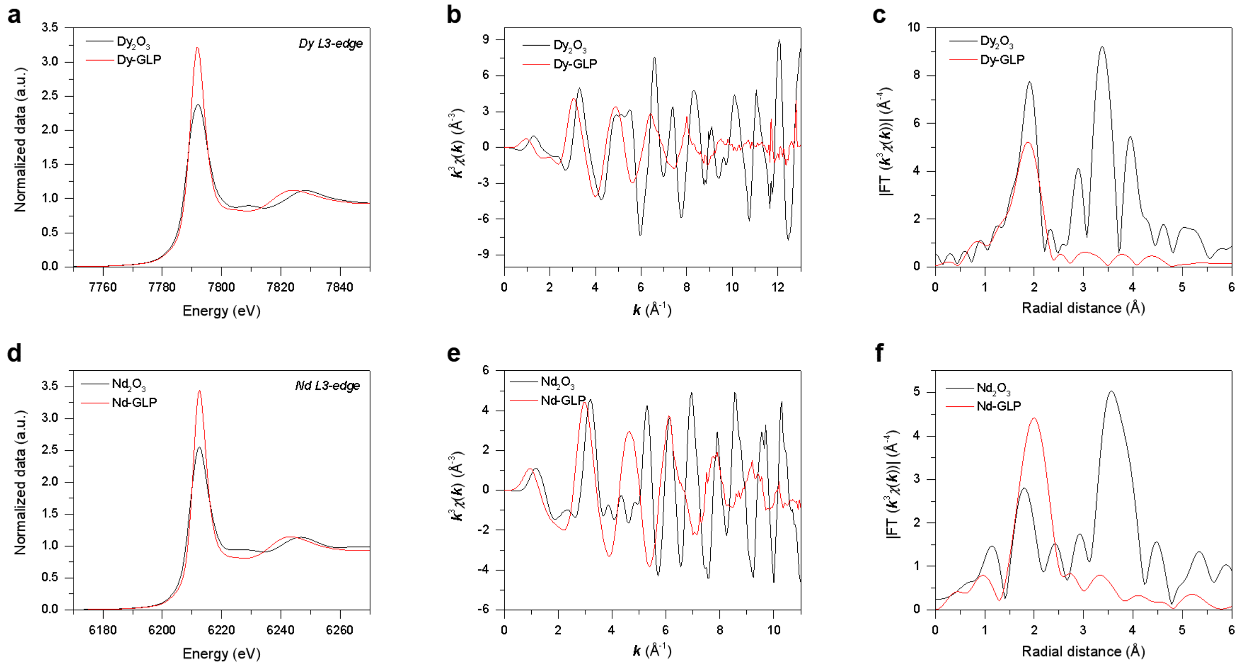

Supplementary Figure 15. Analysis of Dy and Nd L_3-edge XANES spectra. (a,d) Normalized L_3-edge XANES spectra. (b,e) k^3-weighted spectra in *k* space. Measured and fitted spectra are matched very well for both samples. (c,f) Fourier-transform (FT)-EXAFS spectra fittings. R (Å) is the peak location scale for the Fourier-transformed magnitude in the real space.

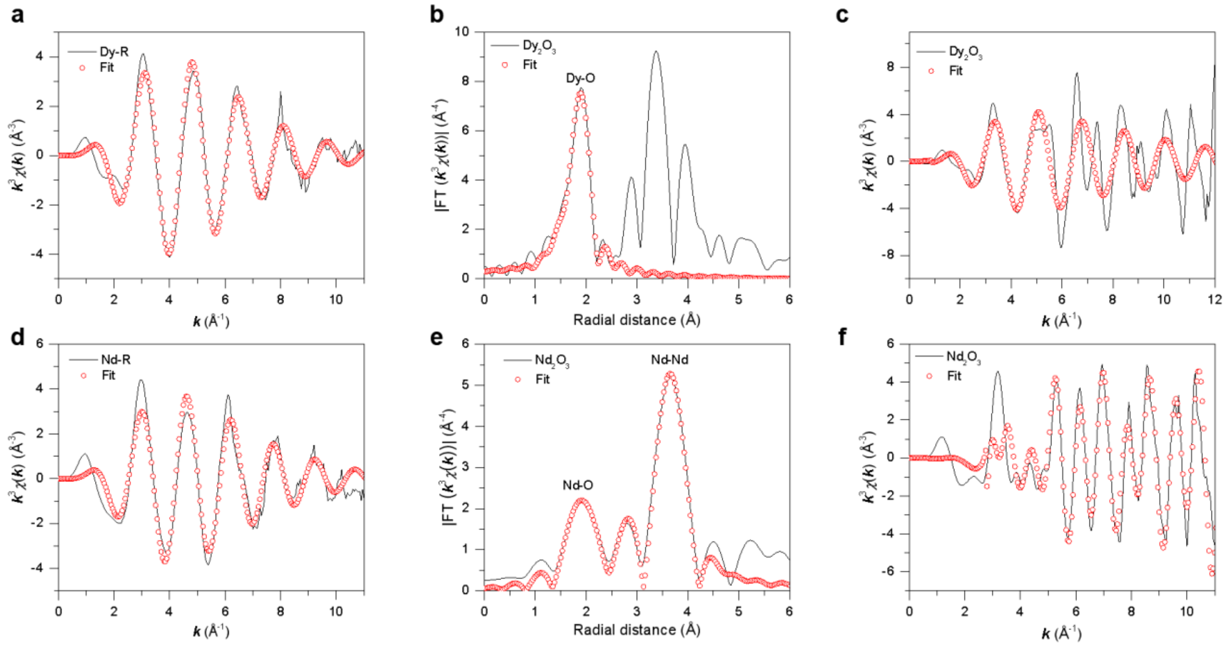

Supplementary Figure 16. Analysis of Dy and Nd L_3-edge XANES spectra. (a,d) k^3-weighted spectra in *k* space for Dy-GLP and Nd-GLP. (b,e) Fourier-transform (FT)-EXAFS spectra fittings. (c,f) k^3-weighted spectra in the *k* space for Dy_2O_3 and Nd_2O_3.

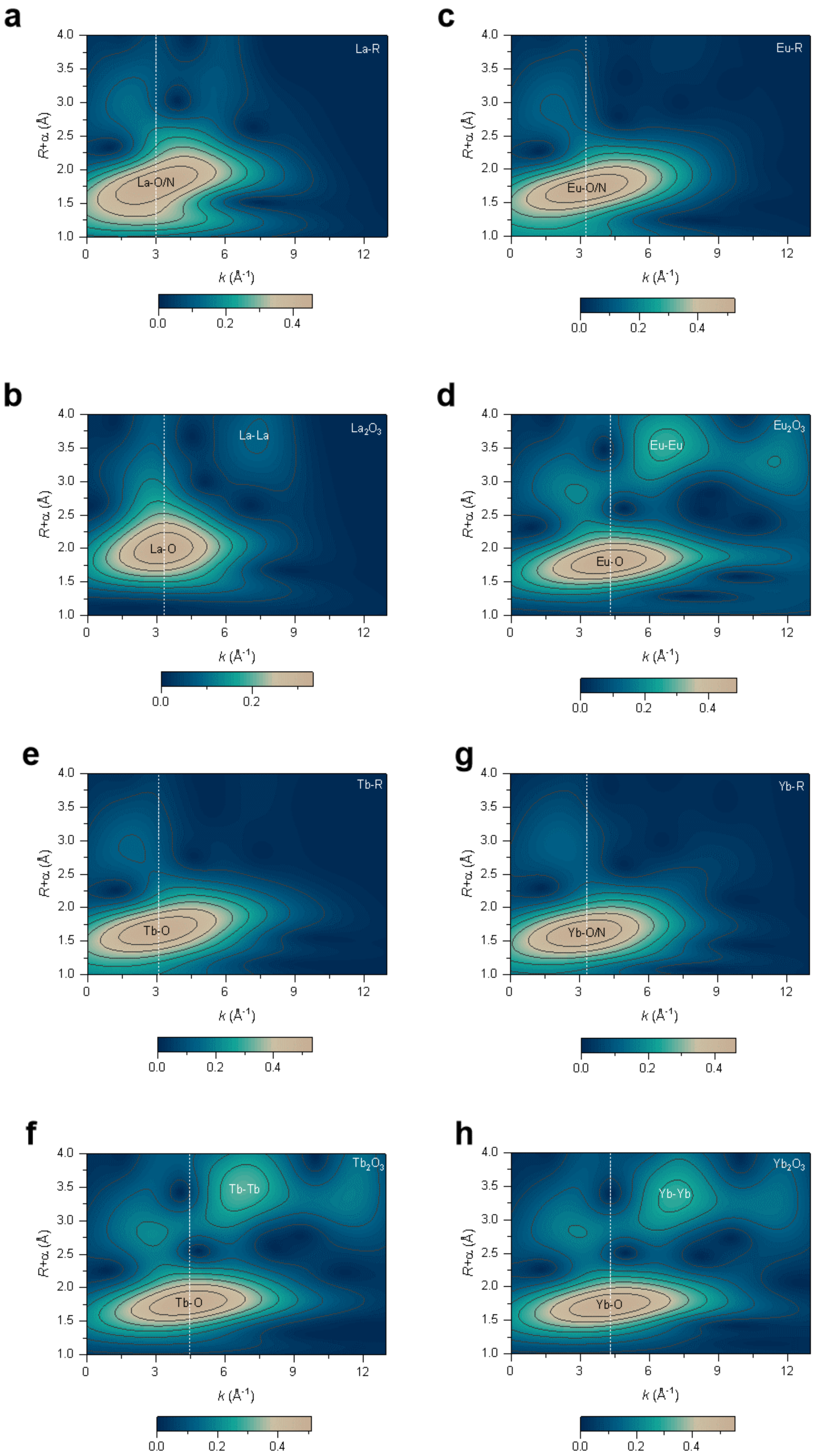
a
La-R
La-O/N
b
La2O3
La-La
La-O
c
Eu-R
Eu-O/N
d
Eu2O3
Eu-Eu
Eu-O
e
Tb-R
Tb-O
f
Tb2O3
Tb-Tb
Tb-O
g
Yb-R
Yb-O/N
h
Yb2O3
Yb-Yb
Yb-O
R+α (Å)
k (Å-1)

Supplementary Figure 17. WT-EXAFS Ln L_3-edge spectra of Ln-GLP and their references. The vertical dashed lines are drawn to guide the eye. α denotes the phase shift. Metallic binds [Ln···Ln] were recorded in the reference samples, whereas these signals were not found in Ln-GLP samples, such as La (a,b), Eu (c,d), Tb (e,f), and Yb (g,h).

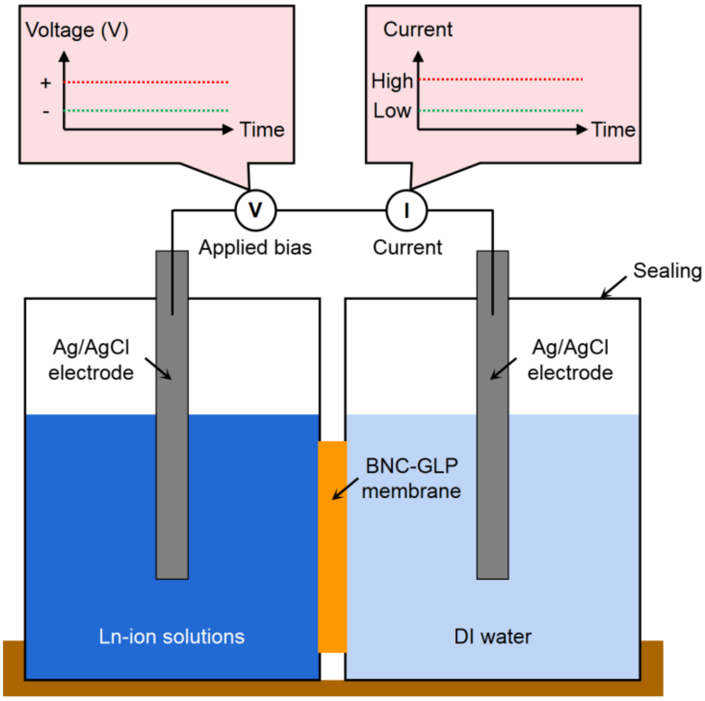

Supplementary Figure 18. Setup of ion permeation through BNC−GLP_{100}. The process driven by an applied bias of +2 V was monitored by a Keithley 6487 picoammeter with real-time current signals.

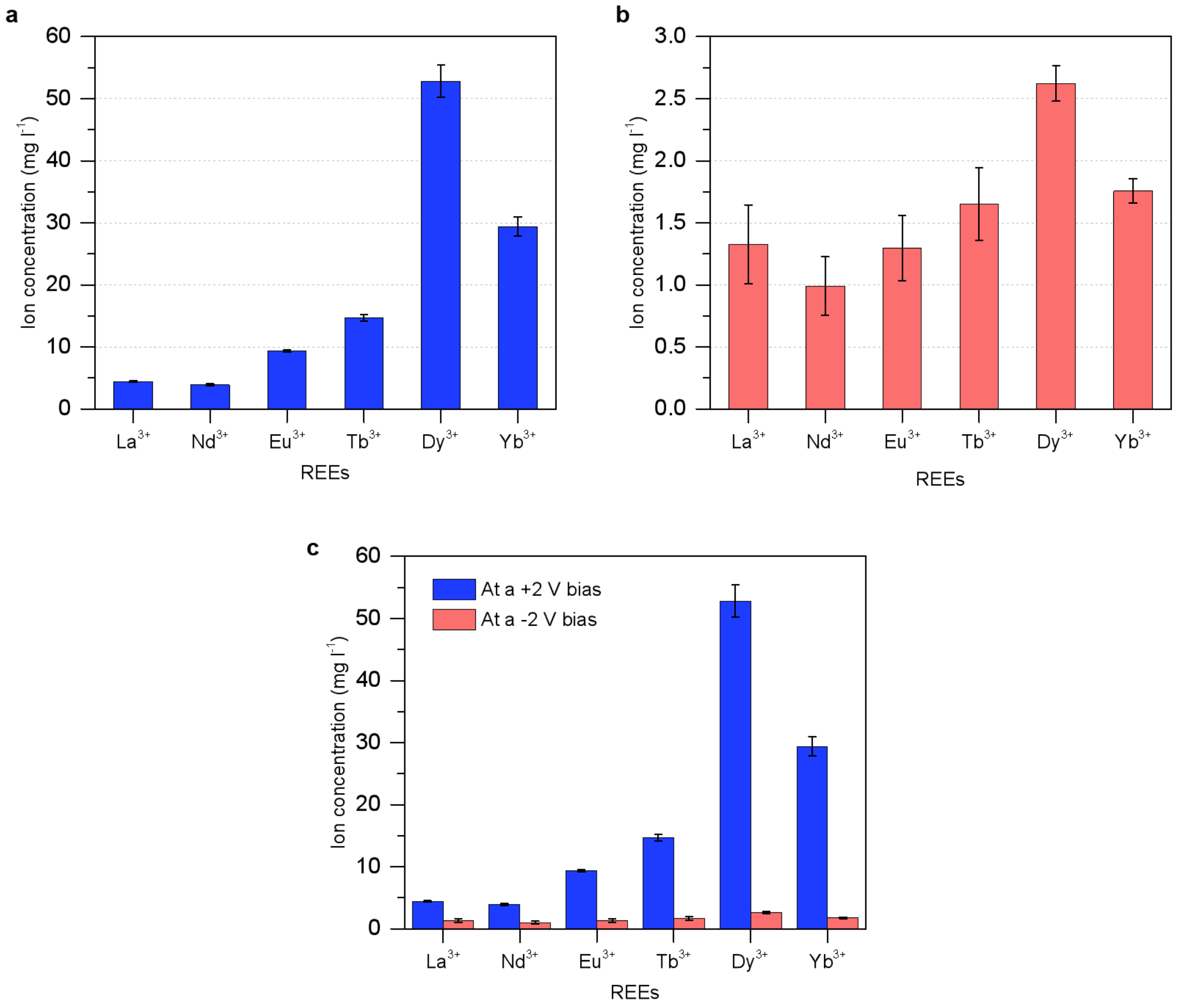

Supplementary Figure 19. Comparison of ion concentration measured by ICP-MS measurements. (a) Ion concentration of the permeate solution by applying a +2 V bias. (b) Ion concentration of the permeate solution by applying a −2 V bias. (c) Comparison of ion concentration at different biases. According these results, it was found that the +2 V bias could drive ion selective diffusion and showed the highest ion concentration for Dy^{3+} ion and the lowest ion concentration for Nd^{3+}, indicating an ultrahigh Dy^{3+}/Nd^{3+} selectivity. On the other hand, the ion concentrations of HREEs are higher than that of LREEs, which suggest that BNC−GLP_{100} enables HREE transport. Error bars give the standard deviation from three independent tests.

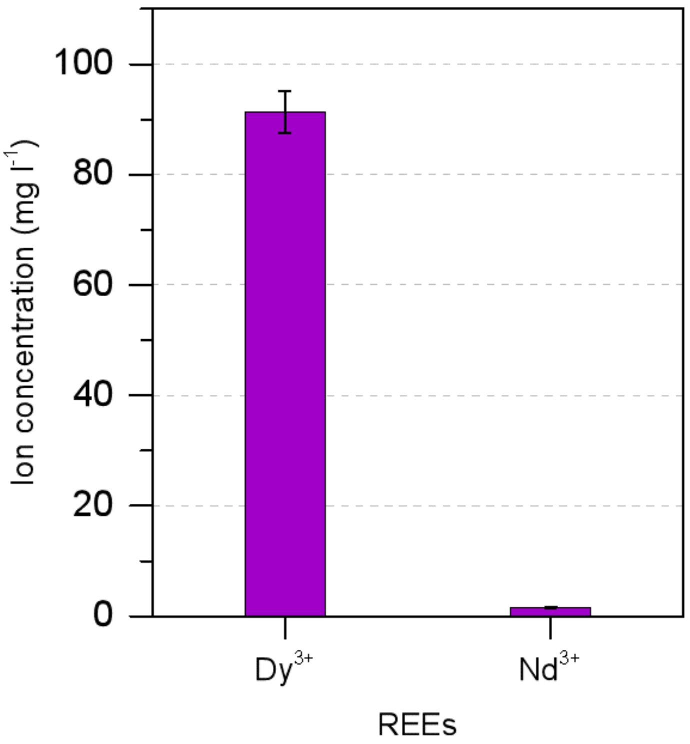

Supplementary Figure 20. Ion concentration of Dy^{3+} and Nd^{3+} in the binary solution. The selectivity of Dy^{3+}/Nd^{3+} was calculated to be 58, according to the ratio of ion concentrations. In the binary solution, the competitive ion transport is far lower than that in the mixture of six REE solutions. Therefore, the selectivity in the binary solution was enhanced. Error bars give the standard deviation from three independent tests.

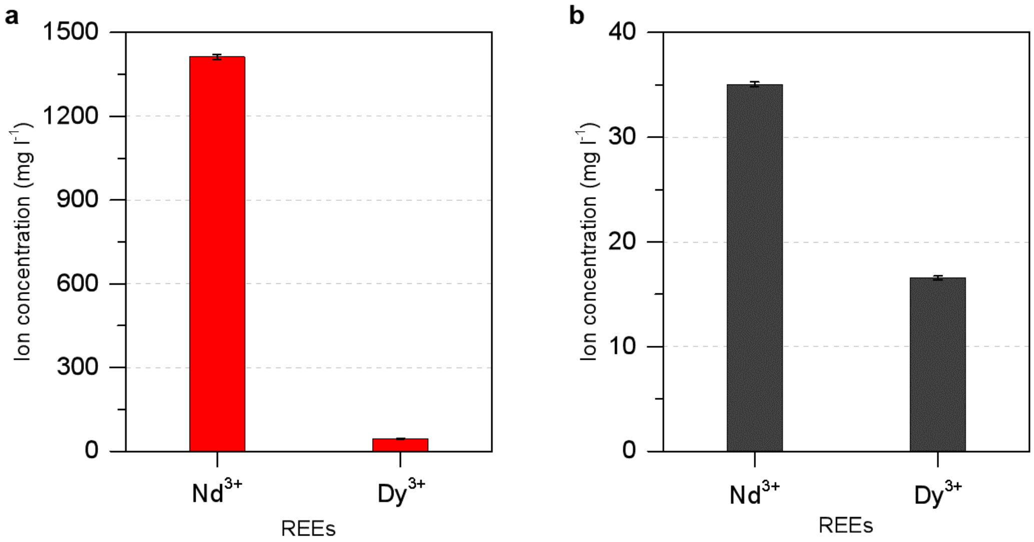

Supplementary Figure 21. The change of ion concentration through BNC−GLP$_{100}$. (a) Ion concentration of the original solution. (b) Ion concentration of the collected solution. In an artificial neodymium magnet system with a 32.3/1 ratio (wt.%) for Nd/Dy, BNC−GLP$_{100}$ could conduct more Dy^{3+} ion through the ion channel. Error bars give the standard deviation from three independent tests.

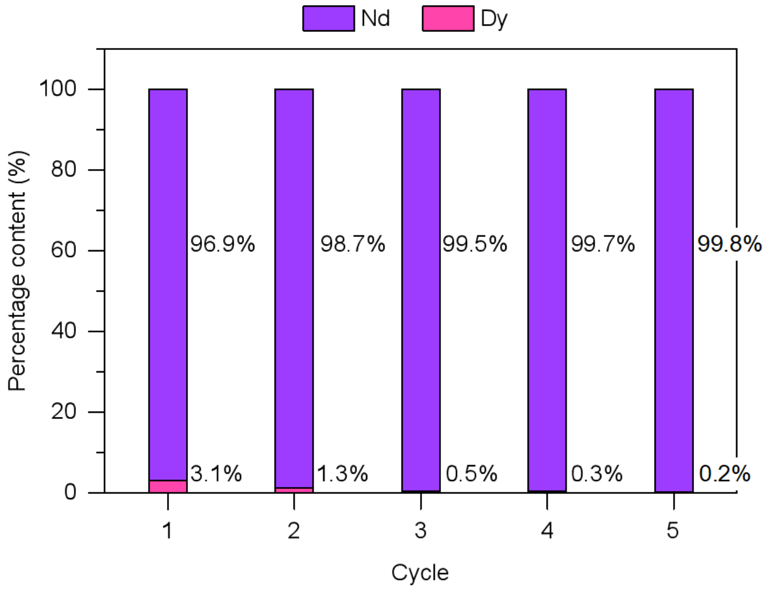

Supplementary Figure 22. Cycle of Dy^{3+} extraction by using the biomimetic nanochannel and the corresponding percentage contents of consecutive cycles. The Dy^{3+} concentration in feed solutions decreased from 3.1 wt.% to 0.2 wt.%, while the Nd^{3+} concentration in feed solutions increased from 96.9 wt.% to 99.8 wt.% after 5 cycles. After further cycling, the concentration remained nearly unchanged (~99.8 wt.% for Nd). It was found that the purification of Nd^{3+} become harder with the increase of cycle, which was attributed to fewer and fewer amounts of Dy^{3+} ion in the feed solution. The result is also theoretically in agreement with the aforementioned reason of the change of selectivity. In conclusion, our system achieved high-performance separation and purification between Dy^{3+} and Nd^{3+} after five cycles. Although high-concentration Nd^{3+} in the feeding solution reduces the selectivity of Dy^{3+} in biomimetic channel after initial separation, ultrahigh Dy/Nd separation can be realized by repeating cycles.

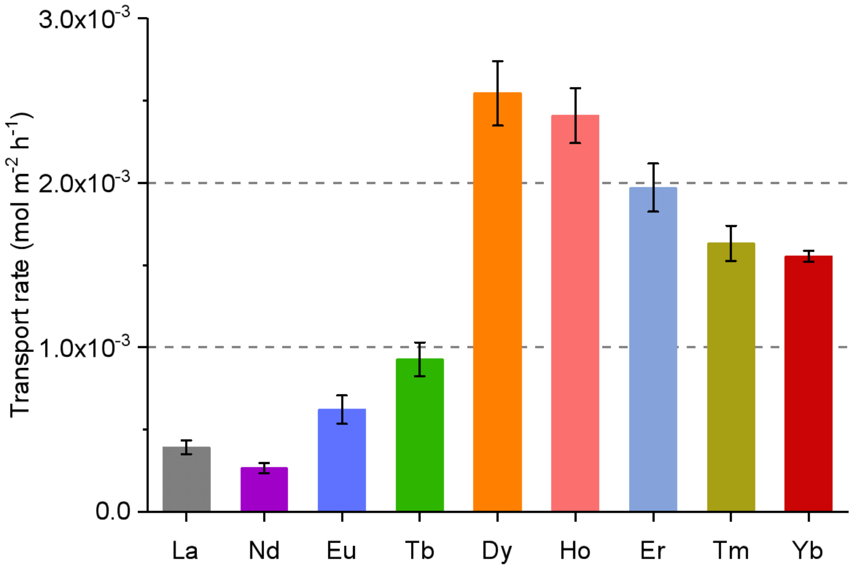

Supplementary Figure 23. Transport rates of other heavy Ln^{3+} ions. The transport rate of Ho^{3+} is similar to that of Dy^{3+}, while the transport rate of Er^{3+} shows a decline. Furthermore, the transport rate of Tm^{3+} significantly drops, approaching that of Yb^{3+}. Error bars give the standard deviation from three independent tests.

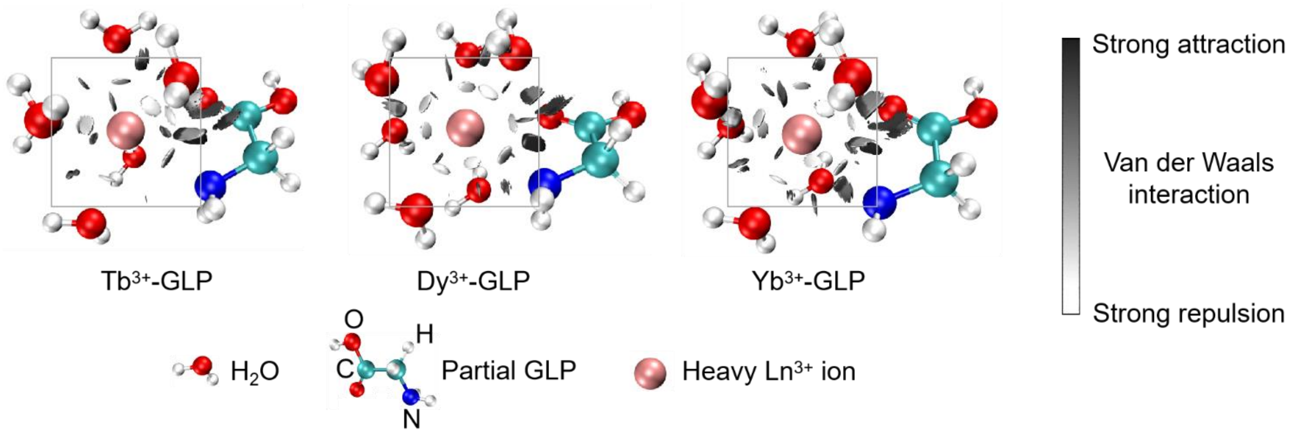

Supplementary Figure 24. Theoretical calculations for visualizing the interactions between heavy Ln^{3+} ions and GLP. The interactions between heavy Ln^{3+} ions and GLP are different. Notably, GLP exhibits a strong binding affinity to Dy^{3+} ion, with an orderly chelation structure. The interactions are evenly distributed around the Dy^{3+} ions, and GLP effectively wraps around Dy^{3+} ion. On the other hand, the binding between Tb^{3+} ion and GLP is weaker, resulting in a disorderly chelation structure with lower and dispersed interactions. Similarly, the interaction between Yb^{3+} ion and GLP shows some improvement, but GLP struggles to wrap around Yb^{3+} ion.

3. Supplementary Tables

Supplementary Table 1. The differences of Ln^{3+} with different coordination numbers on their ion radii.

Entry	Ln^{3+}	Ion radii, Ln^{3+} (pm)		Difference between neighboring Lns (pm)	
		Coordination number (CN)		Coordination number (CN)	
		6	8	6	8
57	La^{3+}	103.2	116.0	\	\
58	Ce^{3+}	102	114.3	1.2	1.7
59	Pr^{3+}	99	112.6	3.0	1.7
60	Nd^{3+}	98.3	110.9	0.7	1.7
61	Pm^{3+}	97	109.3	1.3	1.6
62	Sm^{3+}	95.8	107.9	1.2	1.4
63	Eu^{3+}	94.7	106.6	1.1	1.3
64	Gd^{3+}	93.8	105.3	0.9	1.3
65	Tb^{3+}	92.3	104.0	1.5	1.3
66	Dy^{3+}	91.2	102.7	1.1	1.3
67	Ho^{3+}	90.1	101.5[a]	1.1	1.2
68	Er^{3+}	89.0	100.4	1.1	1.1
69	Tm^{3+}	88.0	99.4	1.0	1.0
70	Yb^{3+}	86.8	98.5	1.2	0.9
71	Lu^{3+}	86.1	97.7	0.7	0.8

[a]Coordination number (CN)=10

Supplementary Table 2. The Ln series divided into light REEs (LREEs) and heavy REEs (HREEs).

☑			☑			☑		☑	☑				☑	
57<br>La<br>138.91	58<br>Ce<br>140.12	59<br>Pr<br>140.91	60<br>Nd<br>144.24	61<br>Pm<br>(145)	62<br>Sm<br>150.36	63<br>Eu<br>151.96	64<br>Gd<br>157.25	65<br>Tb<br>158.93	66<br>Dy<br>162.50	67<br>Ho<br>164.93	68<br>Er<br>167.26	69<br>Tm<br>168.93	70<br>Yb<br>173.05	71<br>Lu<br>174.97
LREEs							HREEs							

Supplementary Table 3. The conduction of Ln^{3+} ions in the bulk solution.

Entry	Diffusion coefficient D ($m^2\ s^{-1}$)	Molar conductivity Λ_m^0 ($S\ cm^2\ mol^{-1}$)	Equivalent conductivity Λ_e^0 ($S\ cm^2\ mol^{-1}$)
**La^{3+}**	0.619	209.1	69.7
**Nd^{3+}**	0.616	208.2	69.4
**Eu^{3+}**	0.602	203.4	67.8
**Tb^{3+}**	0.592[a]	200.1[b]	66.7[c]
**Dy^{3+}**	0.582	196.8	65.6
**Yb^{3+}**	0.582	196.8	65.6

These values of Tb^{3+} are missing, which are presented here using the estimated results according to the lanthanide contraction effect. [a]The value was obtained by calculating the average value of 0.602 and 0.582. [b]The value was obtained by calculating the average value of 203.4 and 196.8. [c]The value was obtained by calculating the average value of 67.8 and 65.6.

Supplementary Table 4. Structure of electronic shell of Ln^{3+} ions.

**Ln^{3+}**	**La^{3+}**	**Nd^{3+}**	**Eu^{3+}**	**Tb^{3+}**	**Dy^{3+}**	**Yb^{3+}**
**Magnetism**	Diamagnetic	Paramagnetic				
**Structure of Ln^{3+}**	**[Xe]**$4f^0$	**[Xe]**$4f^3$	**[Xe]**$4f^6$	**[Xe]**$4f^8$	**[Xe]4**f^9	**[Xe]**$4f^{13}$
J	0	9/2	0	6	15/2	7/2

Supplementary Table 5. EXAFS fitting parameters of Dy_2O_3, Dy−GLP, Nd_2O_3, and Nd−GLP.

Sample	Shell	CN^a	$R(Å)^b$	$\sigma^2(Å^2)^c$	$\Delta E_0(eV)^d$	R factor
Dy L_3-edge (S_0^2=0.858)						
Dy_2O_3	Dy-O	6.0	2.29±0.01	0.0059±0.0014	2.8	0.0208
Dy-GLP	Dy-O	8.7±0.7	2.36±0.01	0.0146±0.00138	-0.1	0.0082
Nd L_3-edge (S_0^2=0.908)						
Nd_2O_3	Nd-O	6.0	2.30±0.03	0.0049±0.0016	1.8	0.0210
	Nd-Nd	8.0	3.59±0.01	0.0078±0.0025	8.8	
	Nd-Nd	3.0	4.15±0.01			
Nd-GLP	Nd-O	8.6±1.8	2.49±0.02	0.0125±0.0031	0.6	0.0042

^{a}*CN*, coordination number; ^{b}*R*, distance between absorber and backscatter atoms; $^c\sigma^2$, Debye-Waller factor to account for both thermal and structural disorders; $^d\Delta E_0$, inner potential correction; *R* factor indicates the goodness of the fit. S_0^2 was fixed to 0.858 and 0.908, according to the experimental EXAFS fit of Dy_2O_3 and Nd_2O_3 by fixing CN as the known crystallographic value. Fitting range: $3.0 \leq k$ ($Å^{-1}$) ≤ 13.2 and $1.5 \leq R$ (Å) ≤ 2.5 (Dy_2O_3); $2.0 \leq k$ ($Å^{-1}$) ≤ 10.1 and $1.1 \leq R$ (Å) ≤ 3.0 (Dy-GLP); $2.0 \leq k$ ($Å^{-1}$) ≤ 10.1 and $1.3 \leq R$ (Å) ≤ 5.0 (Nd_2O_3); $3.0 \leq k$ ($Å^{-1}$) ≤ 10.0 and $1.3 \leq R$ (Å) ≤ 2.7 (Nd-GLP). A reasonable range of EXAFS fitting parameters: $0.700 < S_0^2 < 1.000$; $CN > 0$; $\sigma^2 > 0$ $Å^2$; $|\Delta E_0| < 10$ eV; R factor < 0.02.

Supplementary Table 6. EXAFS fitting parameters of La_2O_3, La−GLP, Eu_2O_3, and Eu−GLP, Tb_2O_3, Tb−GLP, Yb_2O_3, and Yb−GLP.

Sample	Shell	CN^a	R(Å)^b	σ^2(Å^2)^c	ΔE_0(eV)^d	R factor
Eu_2O_3	Eu-O	6.0	2.32±0.01	0.0083±0.0021	1.2	0.0147
	Eu-Eu	3.0	3.61±0.01	0.0065±0.0054		
	Eu-Eu	3.0	4.08±0.02	0.0065±0.0054		
	Eu-Eu	3.0	4.63±0.02	0.0065±0.0054		
Eu-GLP	Eu-O	5.2±0.6	2.41±0.01	0.0021±0.0003	1.5	0.0092
Tb_2O_3	Tb-O	8.0	2.31±0.01	0.0075±0.0014	4.9	0.0175
	Tb-Tb	12.0	3.57±0.01	0.0084±0.0010		
	Tb-O	8.0	4.32±0.02			
Tb-GLP	Tb-O	7.5±0.9	2.39±0.01	0.0143±0.0019	1.9	0.0118
Yb_2O_3	Yb-O	6.0	2.23±0.01	0.0065±0.0009	3.3	0.0116
	Yb-Yb	3.0	3.41±0.03	0.0181±0.0065		
	Yb-Yb	3.0	3.53±0.01			
	Yb-Yb	3.0	3.94±0.01			
Yb-GLP	Yb-O	5.9±0.5	2.28±0.02	0.0083±0.0050	1.1	0.0173
La_2O_3	La-O	6.0	2.59±0.03	0.0188±0.0081	2.7	0.0060
La-GLP	La-O	5.1±0.8	2.55±0.02	0.0163±0.0046	0.1	0.0156

^{a}*CN*, coordination number; ^{b}*R*, distance between absorber and backscatter atoms; $^c\sigma^2$, Debye-Waller factor to account for both thermal and structural disorders; $^d\Delta E_0$, inner potential correction; *R* factor indicates the goodness of the fit. S_0^2 was fixed to 1.0. Fitting range: 3.0 ≤ k (Å^{-1}) ≤ 10.5 and 1.3 ≤ R (Å) ≤ 4.5 (Eu_2O_3); 3.0 ≤ k (Å^{-1}) ≤ 10.0 and 1.0 ≤ R (Å) ≤ 2.5 (Eu-GLP); 3.0 ≤ k (Å^{-1}) ≤ 11.3 and 1.0 ≤ R (Å) ≤ 4.5 (Tb_2O_3); 2.0 ≤ k (Å^{-1}) ≤ 10.2 and 1.2 ≤ R (Å) ≤ 2.7 (Tb-GLP); 3.0 ≤ k (Å^{-1}) ≤ 13.7 and 1.3 ≤ R (Å) ≤ 4.5 (Yb_2O_3); 2.0 ≤ k (Å^{-1}) ≤ 9.0 and 1.1 ≤ R (Å) ≤ 2.5 (Yb-GLP); 2.0 ≤ k (Å^{-1}) ≤ 9.0 and 1.4 ≤ R (Å) ≤ 3.0 (La_2O_3); 1.5 ≤ k (Å^{-1}) ≤ 9.2 and 1.4 ≤ R (Å) ≤ 2.5 (La-GLP). A reasonable range of EXAFS fitting parameters: 0.700 < S_0^2 < 1.000; CN > 0; σ^2 > 0 Å^2; $|\Delta E_0|$ < 10 eV; R factor < 0.02.

Supplementary Table 7. Atomistic coordinates for the mode of the La^{3+}-GLP compound optimized by using the PBE0 method.

Atom	x/a	y/b	z/c
O	-0.32524523	-2.73420702	0.44356798
O	-0.00044491	2.55677337	0.98887602
O	0.55650308	1.64505227	-2.02285821
O	-1.69795717	0.17385328	2.47927114
H	-1.0910073	-2.89882899	1.01474302
H	-0.60226327	-3.07268403	-0.42306509
H	0.96109116	2.59523132	0.86637801
H	-0.10214593	2.36380036	1.93427909
H	1.38695916	1.93448824	-1.61380918
H	-0.02026992	2.42240336	-1.96735821
H	-2.62948423	0.35850135	2.28005612
H	-1.70118422	-0.73046679	2.83169416
O	-1.0994972	-1.14991285	-2.44224624
H	-1.63617021	-0.46040877	-2.86775027
H	-0.24158513	-1.0937409	-2.89400628
C	3.03256416	-0.36105502	-0.16858807
O	2.13020006	-0.86683201	-0.81754111
O	4.26205426	-0.20883008	-0.6393161
H	4.28545924	-0.54210311	-1.55879217
C	2.88794418	0.19210203	1.22369704
H	3.76814223	-0.10144404	1.80840009
H	2.91653824	1.28462211	1.12729203
N	1.62011106	-0.20169493	1.81572308
H	1.67878001	-1.16440001	2.14404911
H	1.44019608	0.37762212	2.63184015
O	-3.01015735	-1.25090775	0.05406295
H	-3.06983738	-1.55366377	0.97342102
H	-2.81494638	-2.05884982	-0.44608209
O	-2.39555813	1.93948646	-0.72656611
H	-1.81341905	2.65264848	-0.41645409
H	-2.35722412	2.00937446	-1.69414618
O	-0.32524523	-2.73420702	0.44356798
La	-0.5715901	-0.0128478	-0.02297206

Supplementary Table 8. Atomistic coordinates for the mode of the Nd^{3+}-GLP compound optimized by using the PBE0 method.

Atom	*x/a*	*y/b*	*z/c*
O	0.11821681	0.64133116	-2.54607127
O	-0.11365922	0.72761415	2.50282012
O	-0.22703801	-2.09817208	1.40264203
O	1.27545875	2.49960639	0.16442894
H	0.99015388	0.56924422	-2.9614993
H	-0.37495317	-0.11227094	-2.9079723
H	-1.04715128	0.55217606	2.69711513
H	-0.02704729	1.68583923	2.61955912
H	-1.16472707	-2.24003716	1.20748502
H	-0.19843702	-1.93294806	2.3570411
H	2.23370281	2.59810347	0.05599893
H	0.89908468	3.05029941	-0.53846012
O	0.95461709	-2.238946	-1.29764617
H	1.91078718	-2.37806093	-1.33966718
H	0.64530012	-2.89996807	-0.65912613
C	-2.88830435	-0.27090615	-0.3025461
O	-1.90145223	-0.88133411	-0.68656513
O	-4.10399441	-0.76480828	-0.3411311
H	-4.07092633	-1.67673235	-0.68177013
C	-2.82722646	1.11075596	0.28226094
H	-3.70588457	1.68264594	-0.05831108
H	-2.95315845	0.98677195	1.37217603
N	-1.54994941	1.73628811	0.00182092
H	-1.56133444	2.17130014	-0.92235415
H	-1.37960745	2.48355218	0.67440998
O	2.85230105	0.21070334	-1.24168517
H	2.95468799	1.08209941	-1.6490502
H	2.95495111	-0.4115727	-1.97570923
O	2.5096411	-0.70985076	1.63561505
H	2.17659012	-1.30913683	2.3189751
H	3.15279919	-1.24533975	1.14853001
Nd	0.54658789	0.01769614	0.04353893

Supplementary Table 9. Atomistic coordinates for the mode of the Eu^{3+}-GLP compound optimized by using the PBE0 method.

**Atom**	***x/a***	***y/b***	***z/c***
O	0.12377806	-1.86316599	1.76977608
O	-0.65081716	1.72223725	-1.50291017
O	1.62628918	-1.91125592	-1.21412214
H	0.55718013	-2.65559603	1.41439606
H	0.61622409	-1.66240195	2.58156915
H	-0.16947314	1.97266529	-2.30618722
H	-0.6659972	2.53056631	-0.96514812
H	2.58733124	-1.80349787	-1.12593814
H	1.4312192	-2.751375	-0.76816811
O	0.5460259	2.18925034	1.37119305
H	1.05948891	2.86201241	0.89693102
H	-0.36243018	2.53093433	1.37624006
C	-2.84968626	0.05340302	0.26837097
O	-1.8803972	0.42981009	0.91830402
O	-4.09149437	0.36377099	0.590088
H	-4.07816039	0.93575203	1.38188206
C	-2.75826721	-0.77515904	-0.98103512
H	-3.56403024	-1.51717313	-0.97663313
H	-2.95534526	-0.093789	-1.81550219
N	-1.42939909	-1.35517802	-1.12860613
H	-1.40500704	-2.27225409	-0.6850331
H	-1.23835706	-1.51019303	-2.11564621
O	2.76114318	-0.05211873	1.32723405
H	3.34626825	-0.65926775	0.84591902
H	2.62188318	-0.48413677	2.18513812
O	2.4133841	1.02479334	-1.41696016
H	2.08637705	1.48757836	-2.20385122
H	2.8175481	1.72296441	-0.87638112
Eu	0.542627	-0.00067583	0.00939395

Supplementary Table 10. Atomistic coordinates for the mode of the Tb^{3+}-GLP compound optimized by using the PBE0 method.

Atom	*x/a*	*y/b*	*z/c*
O	-0.24916674	-2.04785897	-1.46608292
O	0.68420698	1.91507042	1.08940028
O	-1.59874286	-1.77510207	1.46409931
H	-0.91350073	-2.70203407	-1.1927439
H	-0.49444678	-1.82417897	-2.38099299
H	0.41545994	2.07470041	2.00820235
H	0.44839389	2.72737146	0.61845324
H	-2.56151095	-1.68615815	1.3833303
H	-1.40827377	-2.65685212	1.10564028
O	-0.54948011	1.8228693	-1.72233194
H	-1.21235522	2.4847443	-1.47099592
H	0.29625792	2.29406141	-1.69000894
C	2.84353232	-0.07326754	-0.31401183
O	1.84234623	0.09624838	-1.04136789
O	4.09497341	0.03389158	-0.76070986
H	4.07656938	0.2887546	-1.70597193
C	2.75806034	-0.36698657	1.15511728
H	3.56034946	-1.05997855	1.4326813
H	2.95909227	0.57995152	1.66157932
N	1.42233928	-0.83650773	1.51674131
H	1.39615937	-1.8544788	1.46105831
H	1.23406924	-0.60243872	2.48713338
O	-2.7777731	-0.20496005	-1.2379049
H	-3.39906811	-0.64335014	-0.64340386
H	-2.62936703	-0.84762808	-1.94586795
O	-2.30277319	1.29059811	1.28156529
H	-1.89260722	1.92801319	1.88362934
H	-2.78305628	1.83529511	0.63932424
Tb	-0.52010494	-0.01361184	-0.00258781

Supplementary Table 11. Atomistic coordinates for the mode of the Dy^{3+}-GLP compound optimized by using the PBE0 method.

Atom	x/a	y/b	z/c
O	-0.21399661	-1.87535206	-1.63369597
O	0.68026595	1.64694334	1.51179327
O	-1.57299971	-1.88406126	1.20471925
H	-1.01845159	-2.41837921	-1.61366696
H	-0.20828766	-1.48372803	-2.52229504
H	0.27665392	1.68785728	2.39290534
H	0.50634782	2.51830538	1.12119524
H	-2.53291081	-1.73671038	1.21375725
H	-1.45507759	-2.6987563	0.68911321
O	-0.54537622	2.1749372	-1.33372695
H	-1.16071235	2.78516416	-0.89707191
H	0.32417179	2.59588736	-1.24452694
C	2.83409735	0.03381452	-0.30406987
O	1.87415823	0.34747741	-0.99914792
O	4.0749734	0.37445273	-0.59928489
H	4.07291132	0.90085576	-1.42247095
C	2.73162045	-0.75064155	0.97192723
H	3.53488762	-1.4953265	0.99350723
H	2.92405437	-0.04841247	1.78956629
N	1.40062544	-1.32436578	1.11678724
H	1.37446057	-2.24194086	0.67438621
H	1.20369144	-1.47771383	2.10317831
O	-2.70425106	-0.07206928	-1.30008794
H	-3.28781002	-0.6500564	-0.78197791
H	-2.51172497	-0.58569029	-2.101495
O	-2.32105619	1.08084887	1.39152426
H	-1.94615528	1.88234098	1.78975329
H	-2.97939029	1.41566979	0.76060221
Dy	-0.5225619	0.00419304	-0.01069484

Supplementary Table 12. Atomistic coordinates for the mode of the Yb^{3+}-GLP compound optimized by using the PBE0 method.

Atom	x/a	y/b	z/c
O	-0.09188639	-1.81775606	-1.61277611
O	0.67168422	1.54016807	1.57475614
O	-1.53834351	-1.86565883	1.1400411
H	-0.89462454	-2.36202797	-1.66167011
H	-0.02133532	-1.40922104	-2.49139417
H	0.44862116	1.29403609	2.48714821
H	0.24566332	2.40321021	1.44720813
H	-2.50102357	-1.73877266	1.1429961
H	-1.39751963	-2.67954091	0.62875907
O	-0.60057078	2.15446833	-1.26950608
H	-1.40904077	2.62245549	-1.00533906
H	0.12410036	2.71268125	-0.94426005
C	2.80212614	0.09484861	-0.29873201
O	1.83550413	0.4710768	-0.95278005
O	4.04013829	0.45300944	-0.57946003
H	4.03074939	1.05327349	-1.35060709
C	2.70560299	-0.78919344	0.90985509
H	3.49060392	-1.55109762	0.85504109
H	2.92854611	-0.16168043	1.77888016
N	1.3646108	-1.34734026	1.0332661
H	1.32142165	-2.24491232	0.55305606
H	1.16522475	-1.53642124	2.01297317
O	-2.6345423	-0.06721851	-1.30879308
H	-3.22344845	-0.67269046	-0.83028505
H	-2.42626236	-0.53643858	-2.13306615
O	-2.29146709	1.02596752	1.35388612
H	-1.87964999	1.45734149	2.11906118
H	-2.65424001	1.75720163	0.82879208
Yb	-0.50100813	0.00053715	-0.01571198

Supplementary Table 13. Parameters (K_a, n, ΔH_a, $T\Delta S_a$, ΔG_a) of ITC measurements for different REE ions.

	La^{3+}	Nd^{3+}	Eu^{3+}	Tb^{3+}	Dy^{3+}	Yb^{3+}
K_a (M^{-1})	$2.291 \pm 0.004 \times 10^6$	$4.106 \pm 0.003 \times 10^5$	$2.848 \pm 0.063 \times 10^6$	$2.929 \pm 0.008 \times 10^6$	$6.605 \pm 0.036 \times 10^6$	$4.277 \pm 0.028 \times 10^6$
n	1.031±0.008	1.130±0.011	1.336±0.014	1.233±0.008	1.233±0.008	1.435±0.018
ΔH (kJ mol^{-1})	-54.673±1.088	-8.167±0.404	-59.697±1.733	-23.530±0.087	-17.250±0.080	-58.113±1.004
$T\Delta S$* (kJ mol^{-1})	-18.373±1.091	23.873±0.421	-22.857±1.724	13.380±0.094	21.676±0.093	-20.265±0.988
ΔG** (kJ mol^{-1})	-36.301±0.004	-32.040±0.017	-36.840±0.054	-36.910±0.007	-38.926±0.013	-37.849±0.016

*T = 298.15 K.
**$\Delta G = \Delta H - T\Delta S$.

4. Supplementary References

(1) Cotton, S. *Lanthanide and actinide chemistry* (John Wiley & Sons, 2013).

(2) Loble, M. W. *et al*. Covalency in lanthanides. an X-ray absorption spectroscopy and density functional theory study of $LnCl_6^{x-}$ (x=3, 2). *J. Am. Chem. Soc*. 137, 2506–2523 (2015).

(3) Moller, P., Cerny, P. & Saupe, F. *Lanthanides, tantalum, and niobium: mineralogy, geochemistry, characteristics of primary ore deposits, prospecting, processing and applications* (Springer, 2014).

(4) Habenschuss, A. & Spedding, F. H. Coordination (hydration) of rare-earth ions in aqueous chloride solutions from X-ray diffraction. 1. $TbCl_3$, $DyCl_3$, $ErCl_3$, $TmCl_3$, and $LuCl_3$. *J. Chem. Phys*. 70, 2797–2806 (1979).

(5) Habenschuss, A. & Spedding, F. H. Coordination (hydration) of rare-earth ions in aqueous chloride solutions from X-ray diffraction. 2. $LaCl_3$, $PrCl_3$, and $NdCl_3$. *J. Chem. Phys.* 70, 3758–3763 (1979).

(6) Habenschuss, A. & Spedding, F. H. The coordination (hydration) of rare-earth ions in aqueous chloride solutions from X-ray diffraction. 3. $SmCl_3$, $EuCl_3$, and series behavior. *J. Chem. Phys.* 73, 442–450 (1980).
